# Identifying Pathoadaptation in *Pseudomonas aeruginosa* Using Glycopolymer Sensor Arrays

**DOI:** 10.1021/acssensors.5c03694

**Published:** 2025-11-28

**Authors:** Callum Johnson, Kathryn G. Leslie, Sara Franco Ortega, James W. B. Moir, John M. Girkin, Helle Krogh Johansen, Ville-Petri Friman, Clare S. Mahon

**Affiliations:** † Department of Chemistry, 3057 Durham University, Durham DH1 3LE, U.K.; ‡ Department of Biology, 3835 University of York, York YO10 5DD, U.K.; § Department of Physics, 3057 Durham University, Durham DH1 3LE, U.K.; ∥ Department of Microbiology, Faculty of Agriculture and Forestry and Viikki Biocenter, University of Helsinki, Helsinki FI-00014, Finland; ⊥ Department of Clinical Microbiology 9301, Copenhagen University Hospital, 53146 Ringhospitalet, Copenhagen 2100, Denmark; # Department of Clinical Medicine, Faculty of Health and Medical Sciences, University of Copenhagen, Copenhagen N 2200, Denmark

**Keywords:** sensor arrays, differential sensing, glycopolymers, biosensing, bacterial evolution, pathoadaptation, bacterial virulence

## Abstract

In-host bacterial evolution presents a major barrier to effective infection management, driving phenotypic adaptations such as antibiotic resistance and altered virulence. *Pseudomonas aeruginosa*, a key opportunistic pathogen, frequently undergoes rapid evolutionary changes during chronic lung infections, complicating diagnosis and treatment. Current strain typing via whole genome sequencing or selective culturing is costly and time-intensive, and the complex relationship between genetic variations and the resulting phenotype makes clinically relevant pathotypes difficult to identify. Here, we report a cross-reactive, glycopolymer-based fluorescent sensor array capable of directly identifying phenotypic changes related to in-host evolution in *P. aeruginosa*. The sensor array can accurately distinguish phenotypic variations arising from single-gene defects and discriminate clinical isolates with known differences in their evolutionary and pathoadaptive trajectories. Notably, our system is also capable of identifying *P. aeruginosa* isolates as distinct from other bacterial species commonly found in complex polymicrobial lung infections. Our modular platform presents an opportunity to develop sensor arrays that target carbohydrate recognition in a variety of pathogens, offering potential application as a rapid diagnostic tool to inform clinical treatment decisions based on the direct classification of phenotypic profiles.

## Graphical abstract

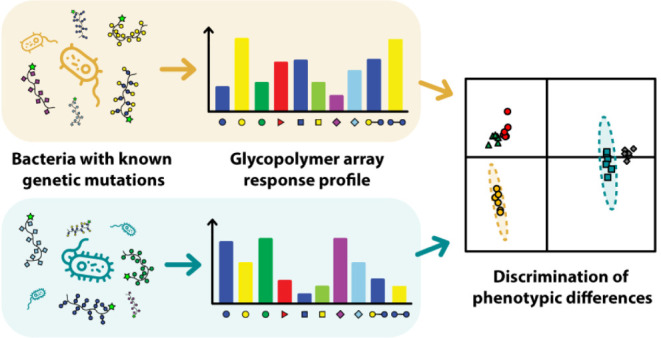

Chronic bacterial infections present huge societal challenges, reducing patient quality of life and placing significant pressures on healthcare systems.^1^ In these cases, bacteria evade the initial response of the host and remain present for extended periods of time—in some cases through asymptomatic infections that may contribute to the dissemination of disease and may be subsequently reactivated within the host or through clinically apparent symptomatic infection.^2^ During these prolonged infections, bacteria may evolve within the host,^3^ leading to persistent infections that are difficult to eradicate or manage. This process of “pathoadaptation” leads to genotypic and phenotypic changes in bacteria, such as altered protein expression, gain or loss of virulence traits, or resistance to antimicrobials, which is forecasted to contribute to 8.2 million annual global deaths by 2050.^4^

*Pseudomonas aeruginosa* is typically associated with chronic respiratory infections in people with cystic fibrosis (pwCF), an autosomal recessive disorder affecting over 160 000 people worldwide,^5^ where deficiencies in the innate immune response lead to extreme vulnerability to lung infections, which severely limit life expectancy (the median age at death of pwCF who died in 2018 was 31 years).^6^ Hospital-acquired *P. aeruginosa* infections are also a frequent cause of mortality for patients undergoing mechanical ventilation.^7^ Initially, *P. aeruginosa* typically displays a nonmucoid phenotype^8^ but rapidly evolves to chronic infective pathotypes characterized by the formation of mucoid alginate biofilms, production of adhesins, antibiotic resistance, and characteristic “loss of function” mutations, including loss of the O-antigen.^9^ These pathotypes are much more difficult to eradicate with antibiotics,^10^ leading to persistent, life-limiting infections.

Current strain-typing protocols are costly and time-intensive, relying on selective culturing methods^11^ or whole genome sequencing.^12^ While diversification within hosts is driven by genetic changes and subsequent selection,^13,14^ the link between genetic variation and the display of functionally different pathotypes is not always clear. A recent longitudinal study focusing on *P. aeruginosa* evolution within immunocompromised patients’ lungs found that different genetic mutations can lead to the same pathoadaptation at the phenotypic level,^15^ making it difficult to predict pathotypes based solely on genetic characteristics. A potential explanation for this observation is that nearly 10% of the *P. aeruginosa* genome encodes regulatory proteins,^16^ providing scope for large phenotypic differences arising from small but significant genetic changes. Therefore, the direct classification of evolving pathogen genotypes based on their virulence characteristics or other phenotypic properties could offer a more robust strategy for rapid diagnostics and efficient treatment compared with genomic information alone.

Many key events in the initiation and progression of bacterial infections are underpinned by molecular recognition at surfaces,^17^ including the attachment of bacteria to cellular surfaces and their adhesion within biofilms.^18^ *P. aeruginosa*, like many other bacteria, produces lectins that interact with carbohydrate motifs displayed on epithelial surfaces and within exopolysaccharide matrices produced during biofilm formation. Several studies have highlighted variation in cellular glycosylation patterns within pwCF, with the predominant display of an asialylated derivative (aGM1) of the mammalian glycolipid GM1 on the epithelial surface, resulting in increased levels of fucose and decreased quantities of neuraminic acid.^19–21^ This variation has been associated with differences in the ability of *P. aeruginosa* to attach to epithelial surfaces via adhesins, a crucial stage in the initial process of colonization. In established infections, cellular surface recognition is likely to be less important than attachment within the biofilm matrix, as suggested by the frequent loss of lectin-bearing pili and flagella within adapted pathotypes.^22^

While the importance of lectins in the establishment and progression of bacterial infection renders them attractive targets for the design of sensors and diagnostics, the development of specific recognition motifs for lectins is complicated by their typically low binding affinities for complementary carbohydrates and their promiscuity in carbohydrate recognition.^17^ The avidity of interactions can be enhanced through the cluster glycoside effect, by attaching multiple copies of the carbohydrate motif to a macromolecular scaffold.^17,23,24^ The cross-reactivity in carbohydrate recognition presents an opportunity to employ array-based sensing methods^25–29^ for the identification of analytes. An array-based approach has been used to screen phenotypic changes in *Staphylococcus aureus* and *Escherichia coli* after exposure to antibiotics.^30^ We recently demonstrated that arrays of fluorescent glycopolymers can discriminate lectins and a selection of pathogenic bacteria through differences in carbohydrate recognition behaviour,^24^ encompassing a broad range of bacteria including *Salmonella enterica*, *Escherichia coli*, vancomycin-sensitive and -resistant strains of *Enterococcus faecium* and *P. aeruginosa* strains PAO1 and PA14. The extension of sensing capability from isolated lectins to bacteria was expected, on account of the prevalence of lectins on bacterial surfaces, and the importance of carbohydrate recognition during infection.^17^ The discrimination of PAO1 and PA14 strains, representing *P. aeruginosa* of moderate and severe virulence, however, was notable, and we decided to explore the discrimination of *P. aeruginosa* isolates of different virulence behaviors, with a view to developing a clinical tool to identify problematic phenotypic changes over the course of prolonged infections.

Here, we describe the synthesis and optimization of a fluorescent glycopolymer sensor array that can discriminate *P. aeruginosa* transposon insertion mutants that differ in their virulence profiles due to mutations in genes associated with several pathoadaptations. We also extend the discriminatory power of the sensor array to differentiate a selection of clinical *P. aeruginosa* CF-lung isolates representing different evolutionary pathoadaptive trajectories, and to discriminate a subset of *P. aeruginosa* CF-lung isolates from other common lung pathogens frequently presented in polymicrobial infections of the CF lung.

## Results and Discussion

The glycopolymers within our sensor array were constructed on a conserved polymer backbone, **P1**, accessed via the reversible addition-fragmentation chain transfer (RAFT) polymerization of 2-hydroxyethyl acrylate (**HEA**) and **M1**, an acrylate derivative bearing an oxime functionality (Scheme 1). **P1** displayed a total degree of polymerization of 80, comprising **HEA** and **M1** in an approximately 1:1 ratio. Kinetic analysis of the polymerization (ESI Section 1.2) demonstrated that both monomers were incorporated at an approximately equal rate, leading to a random copolymer. The ω-trithiocarbonate unit was subjected to aminolysis to generate a thiol, which was immediately reacted with a maleimide-functionalized 4-amino-1,8-naphthalimide, **1**, to generate a fluorescent polymer scaffold, **P2**. The oxime units were then treated with hydrazine hydrate to yield a polymer with pendant acylhydrazide functionalities (**P3**), which were used to install multiple copies of one of ten reducing sugars, generating an array of glycopolymers expected to interact to varying extents with *P. aeruginosa* surface determinants.

**Scheme 1:**
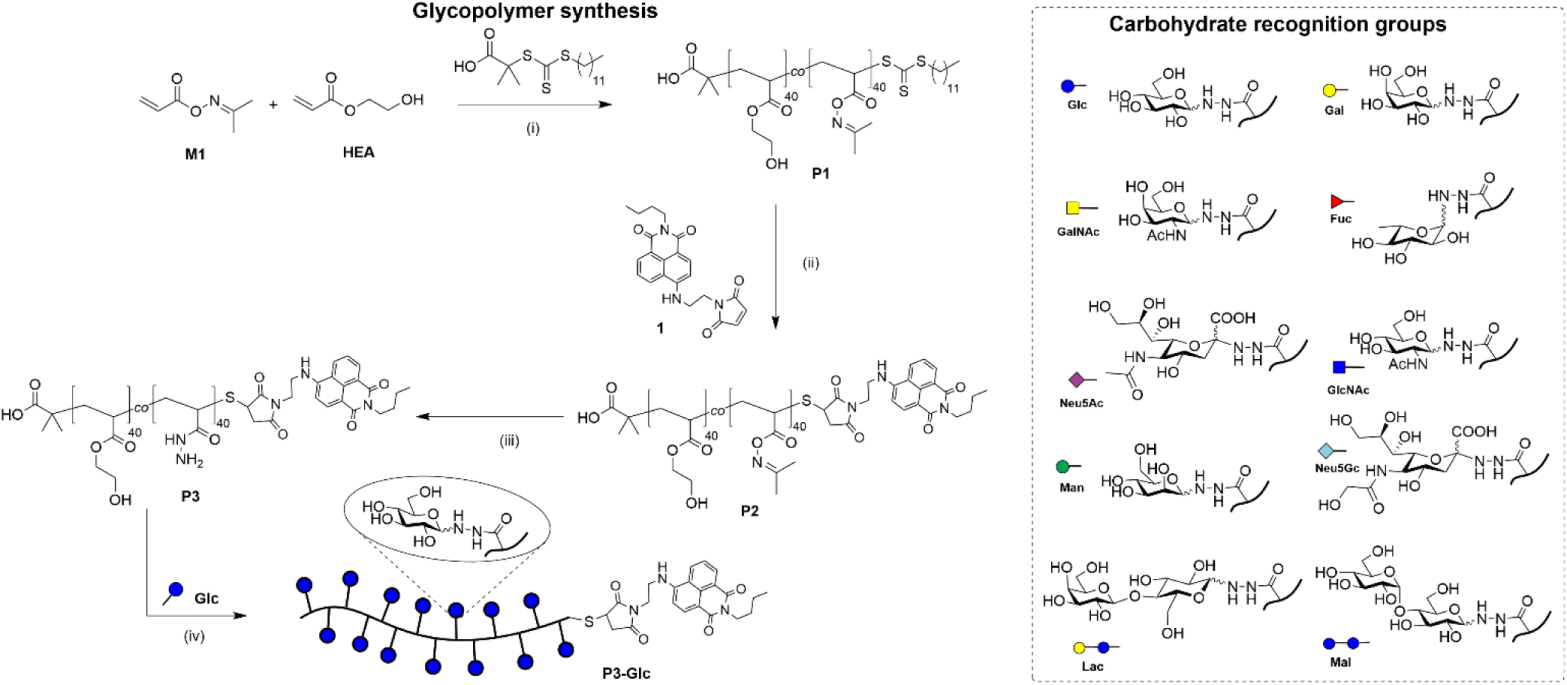
Glycopolymer Synthesis and Carbohydrate Recognition Groups: (i) AIBN, 1,4-Dioxane, 70 °C, 2.5 h; (ii) **1**, Hexylamine, Et_3_N, TCEP, DMF, rt, 18 h; (iii) N_2_H_4_·H_2_O, DMF, 0 °C, 1 h; and (iv) 100 mM NaOAc, Aniline, pH 5.5, 50 °C, 18 h

We first chose to investigate the ability of the sensor array to discriminate a selection of engineered *P. aeruginosa* transposon insertion mutants *in vitro*, with known genetic differences in selected pathoadaptive genes (Table 1). From a library of transposon-insertion mutant strains maintained by Held et al.,^31^ we selected a subset of mutants that display defects in genes associated with virulence: lectin production (PA2570, Δ*lecA*), alginate biosynthesis (PA3542, Δ*alg44*), regulatory protein expression (PA4856, Δ*retS*), and twitching motility (PA4959, Δ*fimX*), as well as PAO1,^16^ representing the complete genome prior to transposon-insertion mutation. Full experimental procedures for the assessment of array response to bacterial addition are available in the ESI (Section 2.1). Briefly, bacteria were grown to saturation (stationary phase) from glycerol stocks in nutrient-rich medium, and then cells were isolated by centrifugation and resuspended in an equivalent volume of phosphate-buffered saline (PBS) at pH 7.4.

**Table 1:** *P. aeruginosa* Genes and Associated Functions

Gene	Associated pathway	Function	Ref(s)
*lecA*	LecA production	LecA is involved in adhesion to host cells via galactose-terminated glycans	^32^
*fimX*	Twitching motility	Regulation of twitching motility in response to environmental cues	^33^
*alg44*	Alginate biosynthesis	Alginate is a structurally important polysaccharide in biofilms	^34^
*retS*	Regulatory protein expression	Modulates phage resistance, biofilm formation, and type IV pili function	^35,36^

Emission spectra were recorded for each receptor within the array (5.0 μM, PBS pH 7.4, 6 replicates), before and after the addition of bacterial suspensions (10 μL aliquot). The integrated emission intensity was used to calculate relative changes in fluorescence emission for each glycopolymer (*I*/*I*_0_). The effect of dilution was accounted for by adding an equivalent volume of buffer to each receptor in the array and dividing receptor *I*/*I*_0_ values by the mean change in the emission intensity for each receptor upon dilution. Upon addition of bacteria to glycopolymers, increases in emission intensity of up to 2-fold were observed with no significant change in emission maximum (Figure 1A,B). 4-Amino-1,8-naphthalimides are highly sensitive to the polarity of their surrounding microenvironment,^37,38^ with enhanced emission in nonpolar media. We propose that upon the binding of glycopolymers to the bacterial surface, the naphthalimide unit resides in a less polar environment than the surrounding aqueous medium, leading to increased emission intensity.

**Figure 1:**
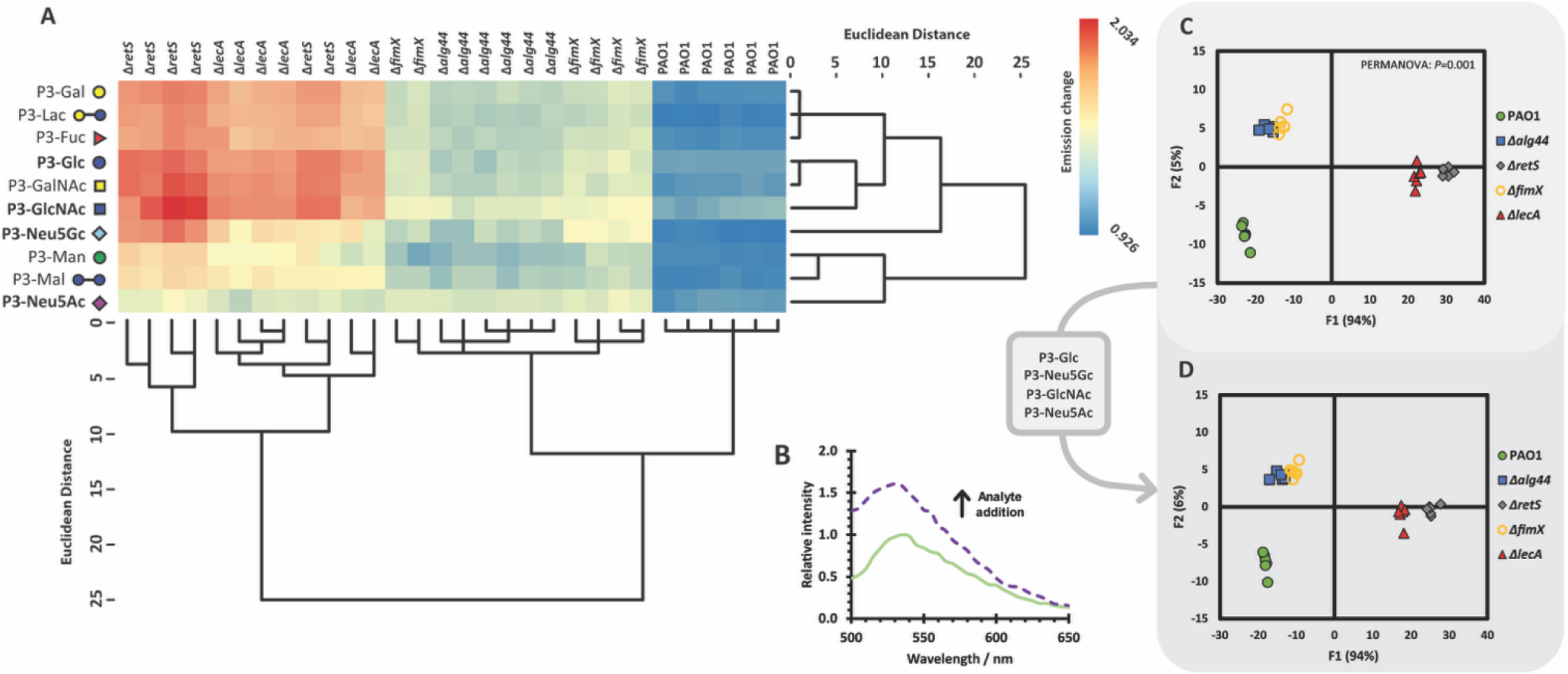
(A) Heat map of fluorescence emission changes for each glycopolymer and bacterial replicate, with dendrograms produced through hierarchical cluster analysis (HCA) of glycopolymer responses to transposon mutants. (B) Representative example of a change in fluorescence emission intensity (λ_ex_ = 450 nm) upon addition of mutant Δ*retS* to **P3-GlcNAc**. (C) Canonical LDA score plot for the analysis of transposon mutants performed in sextuplicate (5.0 μM receptors, pH 7.4). (D) Canonical LDA score plot for the analysis of transposon mutants performed in sextuplicate (5.0 μM receptors, pH 7.4) with a simplified array of glycopolymers, consisting of **P3-Glc**, **P3-GlcNAc**, **P3-Neu5Ac**, and **P3-Neu5Gc**. 100% of original grouped cases were correctly classified, and 100% of cross-validated grouped cases were correctly classified.

The resulting dataset was interrogated using linear discriminant analysis (LDA),^39^ a multivariate statistical tool that analyzes the variance within the dataset and constructs a model that categorizes data points using a combination of linear discriminant functions that describe the responses of the glycopolymers to each analyte. The resulting algorithm can be used to assign unknown analytes to one of these categories. LDA enabled effective discrimination of the mutants, as shown graphically (Figure 1C), with 100% of the variance accounted for by the first and second linear discriminant functions. This analysis enabled classification of the mutants with 100% accuracy. The variance between each pair of clusters identified by LDA was demonstrated to be of statistical significance by PERMANOVA^40^ (Adonis2) (ESI Table S6). The predictive power of the model was confirmed using a “leave-one-out” validation procedure, whereby each data point is systematically excluded from the training set, and the linear discriminant functions calculated using the rest of the data are used to determine its identity. Mutants were identified with a high degree of accuracy (93%) with two discrepancies arising from mutual misclassification of the Δ*fimX* mutant to the Δ*alg44* mutant (ESI Table S7). To further evaluate the predictive ability of the sensor array, the data set was then divided into “training” (4 replicates per analyte) and “unknown” (2 replicates per analyte) datasets (ESI Section 2.7). The “training” dataset was used to construct an LDA algorithm, which was used to assign the “unknown” data points. The “unknown” analytes were assigned with 100% accuracy (Table S10).

To assess the ability of the sensor array to identify bacterial analytes at different effective concentrations, PAO1 was grown to saturation and resuspended in PBS at pH 7.4, as previously described, before these suspensions were further diluted by factors of 1 in 2 and 1 in 4 (ESI Section 2.9). These suspensions were exposed to glycopolymers within the sensor array, as previously described, and assigned as “unknown” analytes using the scoring algorithm established by LDA (Figure 1C, Table S5). In all cases, analytes were scored as PAO1 (Table S13, Figure S5).

To assess whether equivalent discrimination could be achieved using a smaller number of glycopolymers, hierarchical cluster analysis (HCA) was used to identify glycopolymers providing similar response profiles. HCA is a statistical clustering technique that groups data points in a stepwise process based on identifying dissimilarity within the dataset, represented graphically as a dendrogram (Figure 1A). The elimination of glycopolymers displaying similar response profiles allowed us to reduce the number of receptors required for discrimination to four glycopolymers: **P3-Glc**, **P3-GlcNAc**, **P3-Neu5Ac,** and **P3-Neu5Gc**. Subsequent LDA conducted with these four glycopolymers still enabled discrimination of the selected transposon mutants with 100% accuracy (Figure 1D). In a “leave-one-out” validation procedure, mutants were assigned to their groups with 100% accuracy (ESI Table S8), presenting an improvement in predictive performance compared to the complete array of 10 glycopolymers. The hold-out validation procedure described above was repeated, resulting in the assignment of “unknown” analytes with 100% accuracy. This increase in validation scores, while initially unexpected, may reflect that removing the effects of duplication in sensor responses simplifies the features within the dataset that can provide discrimination.^41^

Notably, neuraminic acid-decorated glycopolymers **P3-Neu5Ac** and **P3-Neu5Gc** contribute significantly to the discriminatory power of the sensor array, as indicated by HCA (Figure 1) and PCA loading plots (Figure S4). This observation may reflect the importance of neuraminic acids in cellular recognition,^42^ accounting for a large proportion of the mammalian glycocalyx, with implications for numerous processes of microbial pathogenesis. Alternatively, this behavior may arise as a consequence of the inherent features of the neuraminic acid glycopolymers. Conjugation efficiencies for **Neu5Ac** and **Neu5Gc** were noted to be lower than those for other carbohydrates, resulting in their presence in lower proportions on the polymer backbone (Table S2). This feature may present opportunities for discrimination arising from complementary, less-specific interactions, such as electrostatic effects, in addition to protein–carbohydrate recognition. **P3-Neu5Ac** and **P3-Neu5Gc** are additionally distinct from other glycopolymers in the sensor array in that they are multiply negatively charged, and this difference in electrostatics is likely to amplify the increase in the emission of the naphthalimide probe upon recognition of cell-surface proteins, as the net change in polarity of the medium surrounding the fluorophore upon binding will be greater.^37^

Analytes discriminated by the sensor array display limited genetic differences from one another. Each transposon mutant differs from the reference PAO1 strain by IS*lacZ*/hah or IS*phoA*/hah transposon insertion^31^ in a single gene, disrupting the open reading frames corresponding to the production of a protein associated with a virulence factor or signaling pathway. The effective discrimination of mutants that are genetically similar, yet display different virulence traits, suggests that glycopolymer sensing arrays could find utility in identifying pathoadaptive traits in clinical isolates of *P. aeruginosa* obtained directly from pwCF.^16^

From a library of clinical *P. aeruginosa* isolates obtained during a long-term study on pwCF by Johansen et al.,^43^ we chose five genetically distinct clinical isolates belonging to different “DK” lineages (Pa80 representing DK09, Pa427 representing DK15, Pa202 representing DK28, Pa209 representing DK29, Pa247 representing DK32). These isolates represent different stages of pathoadaptation according to the duration of the infection (0–5.5 years) and are known to display significant genetic and phenotypic differences, having independent evolutionary histories within different patients, and having previously been categorized into distinct genotypes.^43^ The isolate representing the genotype DK09 was identified as having a recent mutation in the *retS* gene, unique among the clinical isolates we tested, which encodes a regulatory protein we previously identified as a key driver of phenotypic changes.^43^ *pilQ*, a gene responsible for transmembrane passage of type IV pili via the PilQ secretin, had undergone mutation in the DK15 and DK32 samples.^44^ Changes to the surface composition of type IV pili may alter glycan-binding properties by affecting the interactions of pili-associated lectins.

Isolates were cultured in nutrient-rich medium and exposed to glycopolymers as detailed previously, resulting in changes to the emission intensity of the naphthalimide probe (Figure 2A), with quenching of emission observed in some cases, in addition to increases in emission intensity, as observed previously. LDA of the changes in emission of the glycopolymers upon exposure to the isolates enabled effective discrimination of the isolates with 100% accuracy, as shown graphically (Figure 2B,C). In this case, 91% of variance within the dataset could be explained by a combination of the first two linear discriminant functions, F1 and F2, accounting for 65% and 26% of variance, respectively (Figure 2B). There was also a non-insignificant contribution to the variance arising from a third function, F3 (6%) (Figure 2C). In a leave-one-out validation assessment, the bacteria were classified with 100% accuracy (ESI Table S19). When the data was split into “training” and “unknown” datasets and assessed as previously described (ESI Section 3.7), the “unknown” analytes were assigned with 90% accuracy, with a single misclassification of a DK32 analyte to DK28 (Table S22). The scoring algorithm established via LDA of this dataset (Figure 2, Table S17) was used to assign diluted suspensions of PAO1, prepared as described earlier. In all cases, analytes were scored as PAO1 (Table S25, Figure S8). Evolution toward decreased virulence but increased drug resistance has been shown to take place during the course of *P. aeruginosa* infection, most significantly during the initial 2–3 years of infection.^15,43^ In LDA analysis, strains sampled earliest in their infection (corresponding with genotypes DK15 and DK28, sampled at first appearance within patient) were more clearly separated from more established strains, which were grouped more closely together (corresponding to DK09, DK29, and DK32 sampled at 1.8, 3.5, and 5.5 years after the first detection of the genotype, respectively). This observation may be consistent with a changing phenotype over time that becomes harder to differentiate as the different bacterial genotypes converge, a hypothesis we intend to examine in detail in a future study. HCA (Figure 2A) and PCA (Figure S7) were again used to find combinations of glycopolymers with similar response profiles, with the aim of reducing the number of sensors required to provide effective discrimination. Again, this analysis enabled the simplification of the array to just four glycopolymers: **P3-Lac**, **P3-Mal**, **P3-GalNAc**, and **P3-Neu5Gc**. Subsequent LDA conducted with these glycopolymers enabled discrimination of the selected isolates with 100% accuracy (Figure 2D,E). Again, utilizing a “leave-one-out” validation procedure, isolates were assigned to their groups with 97% accuracy, with a single discrepancy arising from misclassification of DK32 as DK28 (Table S20). The glycopolymers identified as core to driving discrimination were not entirely identical to those identified as key to discrimination of transposon mutants, although some recognition elements are conserved. Interestingly, of the two neuraminic acid functionalized glycopolymers included within the complete array, **P3-Neu5Gc** was demonstrated to contribute more to discrimination than **P3-Neu5Ac**. *N*-acetylneuraminic acid (**Neu5Ac**) is displayed on human cell surfaces and is known to play a key role as a cellular recognition element and in the evasion of host immune mechanisms, while *N*-glycolylneuraminic acid (**Neu5Gc**) is an analogue which is displayed on cell surfaces of other mammalian cells.^42^ Within pwCF, however, epithelial display of Neu5Ac is reduced as a consequence of display of the asialylated GM1 derivative aGM1.^19^ Moreover, in persistent lung infections, the environment immediately surrounding a bacterium is likely to be dominated by the biofilm: bacteria are primarily exposed to exopolysaccharides and other bacteria, rather than the mammalian epithelial surface itself. **P3-Lac** and **P3-Mal** present terminal galactose and glucose units, respectively, which are displayed within *P. aeruginosa* exopolysaccharide.^45^

**Figure 2:**
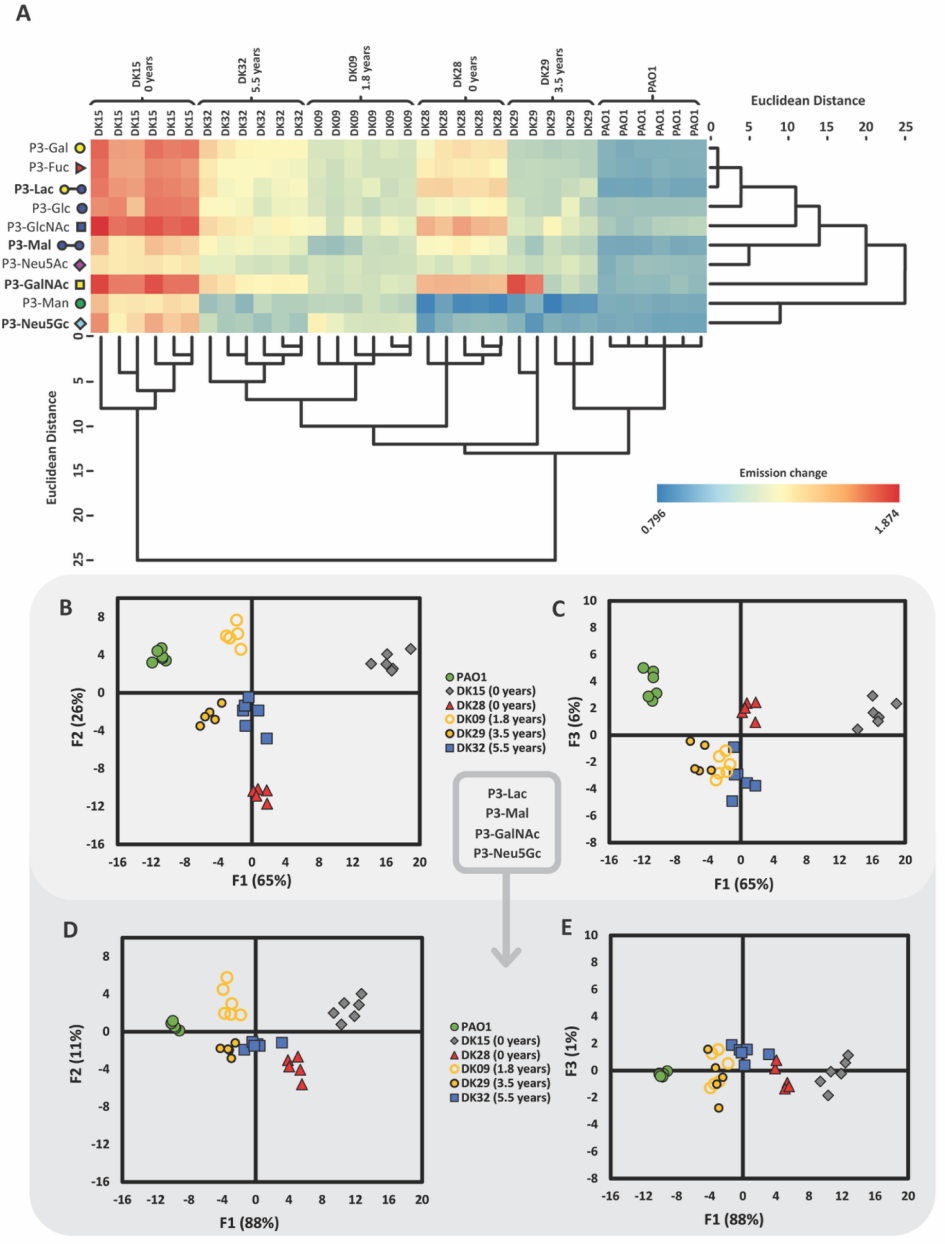
(A) Heat map of fluorescence emission changes for each glycopolymer and replicate with dendrograms produced through hierarchical cluster analysis (HCA) of glycopolymer responses to *Pseudomonas aeruginosa* clinical CF-lung isolates. (B) Canonical LDA score plots for functions 1 & 2 and (C) functions 1 & 3 for the analysis of clinical isolates of known evolutionary lineage performed in sextuplicate (5.0 μM receptors, pH 7.4). 100% of original grouped cases were correctly classified and 94% of cross-validated grouped cases were correctly classified. (D) Canonical LDA score plots for functions 1 & 2, and (E) functions 1 & 3 for the analysis of clinical isolates of known evolutionary lineage performed in sextuplicate (5.0 μM receptors, pH 7.4) with a simplified array of glycopolymers, consisting of **P3-Lac**, **P3-Mal**, **P3-GalNAc**, and **P3-Neu5Gc**. 100% of original grouped cases were correctly classified, and 97% of cross-validated grouped cases were correctly classified.

The ability to rapidly discriminate *P. aeruginosa* strains of different, clinically relevant evolutionary lineages would present a major advantage for the monitoring of persistent infections, enabling improved clinical decision-making. *P. aeruginosa* infections within immunocompromised patients, however, are typically a single component of a complex, multispecies infection,^46,47^ although *P. aeruginosa* is generally considered to be responsible for the majority of irreversible lung tissue damage.^48^ We therefore assessed the ability of the array to identify *P. aeruginosa* pathotypes among a selection of other bacteria typically present within the CF lung: *Staphylococcus aureus*, *Serratia* spp., *and* *Stenotrophomonas maltophilia*. *S. aureus* is a Gram-positive bacterial pathogen, which is frequently implicated in polymicrobial respiratory infections in pwCF.^49^ *S. maltophilia* is also commonly coidentified in pwCF colonized with *P. aeruginosa* ^50,51^ and may transition to a similar mucoid phenotype to that displayed by *P. aeruginosa*. *S. maltophilia* isolates have been misidentified as *Pseudomonas*,^52^ with selective culturing methods or metabolic studies developed to enable identification.^11^ *Serratia* spp. is a Gram-negative bacterium which is increasingly implicated in hospital-acquired infections, including respiratory infections in immunocompromised patients.^53^

The ability of the sensor array to discriminate these lung pathogens was first established by comparing the array’s response to *S. aureus*, *S. maltophilia*, *Serratia*, and PAO1 as a representative *P. aeruginosa* isolate. Using the same experimental procedure as previously employed, lung pathogens were grown in nutrient-rich medium before resuspension in an equivalent amount of buffer. Glycopolymers within the array (5.0 μM, 6 replicates) were then exposed to the bacteria (10 μL aliquots), and LDA of the changes in emission of the probes upon exposure to the isolates (*I*/*I*_0_) enabled effective discrimination of each genus with 100% accuracy (Figure 3A,B). “Leave-one-out” cross-validation showed classification of each species with 92% accuracy, with two mutual misclassifications of *S. maltophilia* and *S. aureus* (ESI Table S31). Data were subsequently split into “training” and “unknown” datasets and assessed as previously described (ESI Section 4.6). In this case, the “unknown” analytes were assigned with 88% accuracy, with a single misclassification of *S. aureus* to *S. maltophilia* (Table S33). The discriminatory power of the sensor array at varying effective concentrations of analytes was again probed using “unknown” analytes consisting of PAO1 grown to saturation, resuspended in PBS, and diluted by factors of 1 in 2 and 1 in 4 as previously described. Upon scoring with the algorithm generated by LDA (Figure 3A,B; Table S29), these analytes were identified as *S. maltophilia* (Table S35), highlighting a limitation of the array in identifying analytes at varying effective concentrations. This difficulty likely arises because the changes in naphthalimide emission observed upon exposure to the range of pathogens are smaller than those observed upon exposure to *P. aeruginosa* analytes, rendering pattern recognition more challenging. This difference in response reflects the design principles of our sensor array: to best enable discrimination of *P. aeruginosa*strains we have focused on incorporating carbohydrate–protein interactions that are important within the evolutionary trajectory of this pathogen. In order to improve the sensor array’s ability to discriminate different respiratory pathogens at different effective concentrations, the receptor pool should be widened to encompass other respiratory disease-relevant processes of molecular recognition to elicit a greater response upon exposure to analytes.

**Figure 3:**
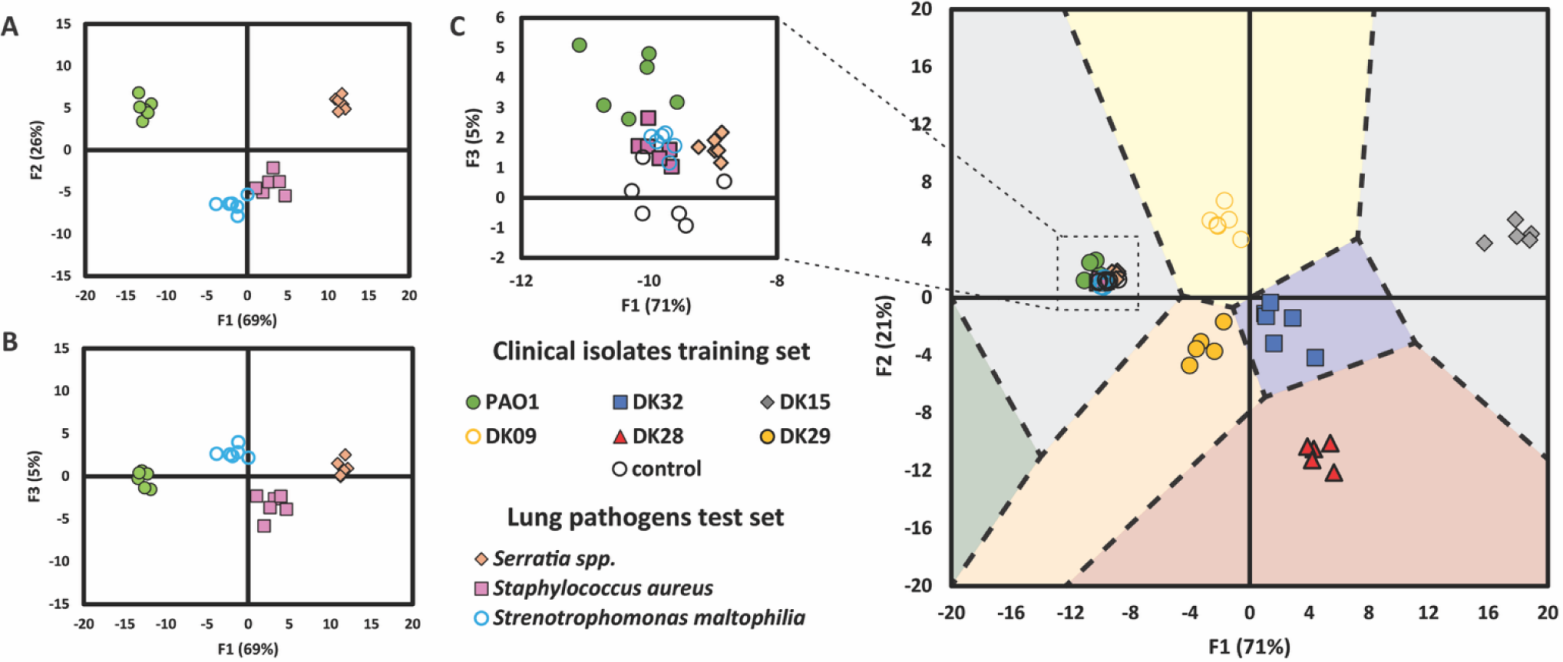
(A) Canonical LDA score plots for functions 1 & 2 and (B) functions 1 & 3 for the analysis of lung pathogens performed in sextuplicate (5.0 μM receptors, pH 7.4). 100% of original grouped cases were correctly classified with 96% of cross-validated grouped cases correctly classified. (C) Territorial map with LDA scores for *P. aeruginosa* CF-lung isolates, including a control and several lung pathogens (*Serratia* spp., *Staphylococcus aureus*, and *Stenotrophomonas maltophilia*), unknown to the LDA model (5.0 μM receptors, pH 7.4). A partial 3D plot showing the third scoring function, F3, is supplied in the ESI (Figure S9).

To explore the predictive power of the glycopolymer sensor array in identifying *P. aeruginosa* isolates as distinct from other lung pathogens, a training dataset was constructed using the response of the complete array to the *P. aeruginosa* clinical isolates tested above (DK09, DK15, DK28, DK29, DK32), PAO1, and a “control” sample consisting of an aliquot of PBS buffer. LDA analysis of this dataset was used to construct a scoring algorithm, which was used to assign the lung pathogen analytes to the most relevant category. The three lung pathogens were all subsequently classified within the “control” group (18/18), with score functions located within the boundaries of this category (Figure 3C; Figure S9). This analysis demonstrates that the glycopolymer sensor array can identify a bacterial infection as either *P. aeruginosa* or an alternative pathogen, in addition to enabling the discrimination of common CF lung pathogens and of *P. aeruginosa* mutants.

## Conclusions

We have described a glycopolymer sensor array comprising a conserved polymer scaffold, 10 carbohydrate recognition motifs, and a fluorescent reporting group, which can identify phenotypic variation in virulence profiles within *P. aeruginosa*, an opportunistic pathogen associated with persistent respiratory infections in immunocompromised patients. The sensor array can identify transposon-insertion mutants with differences in genes associated with pathoadaptive traits, including adhesin production, alginate biosynthesis, and motility. The sensor array was also demonstrated to classify clinical isolates of *P. aeruginosa* representing different evolutionary lineages, displaying different degrees of pathoadaptation and virulence traits. Through detailed statistical analysis of the array’s response, the number of sensors required to achieve effective discrimination can be reduced to just four, streamlining the process of identification and presenting a route to multiplexed combinations of sensors.

Our sensor array was also shown to be capable of identifying *P. aeruginosa* isolates as distinct from other, similar bacteria commonly associated with persistent infections in pwCF—an important distinction considering that *P. aeruginosa* is thought to be responsible for the majority of irreversible tissue damage in polymicrobial infections.^48^ This system could provide the underpinning technology for the development of diagnostic platforms to discriminate *P. aeruginosa* pathotypes and identify virulence features within complex polymicrobial infections, informing treatment decisions. Moreover, given the ubiquity of carbohydrate recognition processes within many key processes of disease, our modular design presents a route to the development of glycopolymer sensor arrays for a host of other bacterial or viral pathogens.

## Supplementary Material



## Data Availability

Polymer characterization data, raw spectroscopic input data and statistical models may be downloaded at http://doi.org/10.15128/r1mp48sc83w.

## Notes

Synthetic details, characterization, raw data, statistical analysis (PDF)
