# Supplementary material for: Identifying Pathoadaptation in Pseudomonas aeruginosa Using Glycopolymer Sensor Arrays
Source: ACS Sens. 2025 Nov 28;11(1):707–15. doi: 10.1021/acssensors.5c03694 (PMC12836338; doi:10.1021/acssensors.5c03694)
Supplement: Supplementary file 1 [file se5c03694_si_001.pdf]

Identifying pathoadaptation in *Pseudomonas aeruginosa* using glycopolymer sensor arrays

Callum Johnson,[a] Kathryn G. Leslie,[a] Sara Franco Ortega,[b] James W. B. Moir,[b] John M . Girkin,[c] Helle Krogh Johansen,[e,f] Ville-Petri Friman,[b,d] and Clare S. Mahon[a*]

a. Department of Chemistry, Durham University, Durham DH1 3LE, U.K.
b. Department of Biology, University of York, York YO10 5DD, U.K.
c. Department of Physics, Durham University, Durham DH1 3LE, U.K.
d. Department of Microbiology, Faculty of Agriculture and Forestry and Viikki Biocenter, University of Helsinki, Helsinki FI-00014, Finland
e. Department of Clinical Microbiology 9301, Ringhospitalet, Copenhagen University Hospital, Copenhagen 2100, Denmark.
f. Department of Clinical Medicine, Faculty of Health and Medical Sciences, University of Copenhagen, Copenhagen N 2200, Denmark

Table of Contents

1. Synthesis and characterisation 3

1.1 Synthetic methods 3

1.1.1 General experimental details 3

1.1.2 Instruments and Analysis 3

1.1.3 Synthesis of P1 3

1.2 P1 Copolymerisation kinetics 5

1.3 Characterisation of P1 6

1.4 Characterisation of P3-carbohydrate 6

1.5 Emission spectra of P3-carbohydrate 7

2. Discrimination of transposon-insertion mutants 8

2.1 Method 8

2.2 Transposon-insertion mutant details 9

2.3 Emission change data 9

2.4 LDA canonical discriminant function coefficients 10

2.5 PERMANOVA analysis 10

2.6 Confusion matrices 11

2.7 Hold-out cross validation 11

2.8 Principal component analysis (PCA) 13

2.9 LDA including PAO1 dilutions 13

3. Discrimination of clinical isolates 15

3.1 Method 15

3.2 Clinical isolate details. 15

3.3 Emission change data 15

3.4 LDA canonical discriminant function coefficient 16

3.5 PERMANOVA analysis 17

3.6 Confusion matrices 18

3.7 Hold-out cross validation 18

3.8 Principal component analysis (PCA) results 20

3.9 LDA including PAO1 dilutions 21

4. Discrimination of lung pathogens 22

4.1 Method 22

4.2 Emission change data 23

4.2.1 Lung pathogen data 23

4.2.2 Clinical isolate with 'unknown' lung pathogens data 24

4.3 LDA canonical discriminant function coefficients 26

4.4 PERMANOVA analysis 26

4.5 Confusion matrix 26

4.6 Hold-out cross validation 26

4.7 3D plot of LDA from Figure 3 28

4.8 Principal component analysis (PCA) results 28

4.9 LDA including PAO1 dilutions 29

5. NMR Spectra 30

6. References 41

1. Synthesis and characterisation

1.1 Synthetic methods

1.1.1 General experimental details

Dialysis membrane (regenerated cellulose with a 3500 molecular weight cut-off) was obtained from Spectrum Dialysis. RAFT chain transfer agent DDMAT was synthesised according to a literature method.[1] All other chemicals were synthesised or purchased from Sigma Aldrich, Fischer Scientific, Biosynth or Fluorochem and used as received unless otherwise stated. Hydroxyethyl acrylate (HEA) (10 mL) was dissolved in H_2O (60 mL) and washed with hexane (4 × 60 mL). The aqueous layer was saturated with NaCl and HEA extracted into EtOAc (60 mL), dried with sodium sulphate and evaporated to dryness. HEA was also passed though basic alumina prior to use.

1.1.2 Instruments and Analysis

NMR spectra were recorded on a Bruker DRX-400 MHz spectrometer (at operating frequencies of 400.13 MHz for ^{1}H, 100.62 MHz for ^{13}C) using commercially available deuterated solvents ($CDCl_3$ (δH = 7.26 ppm), MeOD-d_4 (δH = 3.31 ppm) and D_2O (δH = 4.79 ppm)). Gel permeation chromatography measurements were conducted using an Agilent 1260 instrument equipped with differential refractive index detector and a pair of PL gel 5µm Mixed-D columns (300 x 7.5 mm) with a guard column (Polymer Laboratories Inc.), connected in series. Chromatography was performed in THF (1.0 mL/min) at 35 °C. Near monodisperse poly(methyl methacrylate) standards (Agilent) were used for calibration.

Electrospray ionisation mass spectra (ESI-MS) were obtained on a TQD mass spectrometer with an Acquity UPLC and Acquity photodiode array detector for absorbance data; acetonitrile was used as the solvent. Ultraviolet-Visible (UV-Vis) and fluorescence spectra were obtained using a Tecan SPARK® multimode microplate reader, or a Tecan Infinite 200 Pro multimode plate reader.

Melting points of solid crystalline products were measured using a Cole-Parmer® Stuart MP-200 Digital Melting Point Apparatus.

1.1.3 Synthesis of P1

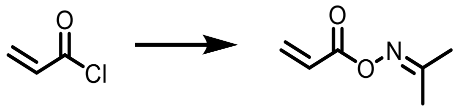

M1

Acetone oxime acrylate[2] (**M1**)

Procedure adapted from literature.[2] Acetone oxime (8.10 g, 110 mmol) and triethylamine (11.2 g, 110 mmol) were dissolved in chloroform (80 mL) and cooled to 0 °C. Acryloyl chloride (8.70 g, 110 mmol) in chloroform (40 mL) was added dropwise to the reaction mixture, which was stirred for 3 h at room temperature. The reaction mixture was then washed with water (3 × 80 mL) and saturated $NaHCO_3$ solution (3 × 80 mL) before being dried over Na_2SO_4. The solvent was removed *in vacuo* to afford **M1** as a pale yellow oil (3.4 g, 24%). δ_H (400 MHz, $CDCl_3$) 2.07 (6 H, d, C(C*H*$_3$)$_2$), 5.93 (1 H, dd, C*H*), 6.22 (1 H, dd, C*H*), 6.54 (1 H, dd, C*H*). δ_C (101 MHz, $CDCl_3$) 17.00, 22.04, 126.68, 131.70, 163.69, 164.42. *m/z* (ESI) 128.15 ((M+H)$^+$, 100%). All data is consistent with that previously reported in literature.[2]

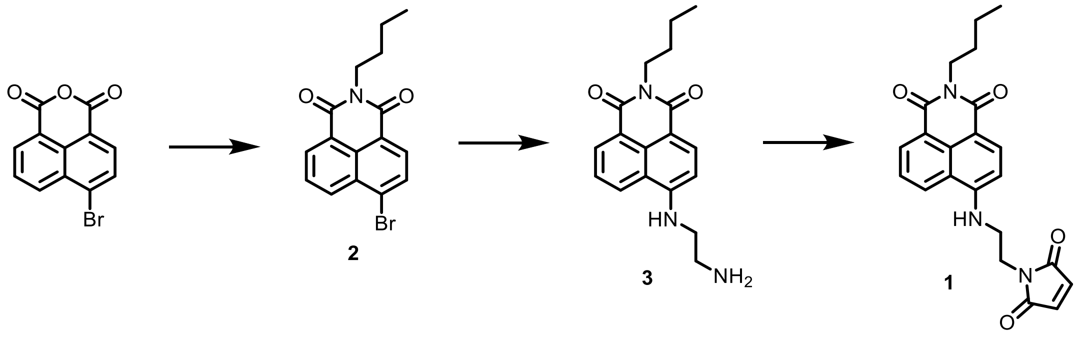

N -Butyl-4-bromo-1,8-naphthalimide[3] (**2**)

Procedure adapted from literature.[3] *N*-Butylamine (0.78 mL, 79 mmol) was added dropwise to 4-bromo-1,8-napthalic anhydride (2.15 g, 850 mmol) suspended in EtOH (70 mL) and heated under reflux for 16 h. After cooling, the solution was poured over an ice-water slurry, and the resulting yellow-brown precipitate was isolated by filtration. The crude product was recrystalised from EtOH to afford **2** as a yellow solid (1.24 g, 52%). δ_H (400 MHz, $CDCl_3$) 1.00 (3 H, t, C*H*3), 1.47 (2 H, m, C*H*2CH3), 1.68 – 1.80 (2 H, m, C*H*2CH2CH3), 4.16 – 4.24 (2 H, m, NC*H*2), 7.87 (1 H, dd, C*H*), 8.07 (1 H, d, C*H*), 8.44 (1 H, d, C*H*), 8.59 (1 H, dd, C*H*), 8.68 (1 H, dd, C*H*). δ_C (101 MHz, $CDCl_3$) 13.84, 20.38, 30.17, 40.38, 122.28, 123.14, 128.04, 128.95, 130.13, 130.58, 131.06, 131.15, 131.96, 133.15, 163.55, 163.58. *m/z* (ESI) 332.25 ((M(^{79}Br)$^+$, 92%), 334.26 ((M(^{81}Br)$^+$, 100%). All data is consistent with that previously reported in literature.[3]

N-Butyl-4-ethylenediamine-1,8-naphthalimide[4] (**3**)

Procedure adapted from literature.[4] Ethylenediamine (14.6 mL, 220 mmol) was heated to 65 °C before the addition of **2** (1.09 g, 1.5 mmol) and the resulting solution was stirred for 3 h. Water (30 mL) was added before extraction into CH_2Cl_2 (40 mL). The organic layer was then washed with water (40 mL) before drying over Na_2SO_4. The crude product was recrystallised from toluene to afford **3** as a yellow solid (0.97 g, 95%). δ_H (400 MHz, $CDCl_3$) 1.00 (3 H, t, C*H3*), 1.47 (2 H, m, C*H2*CH3), 1.71 (2 H, m, C*H2*CH2CH3), 3.20 (2 H, t, NHC*H2*), 3.41 – 3.49 (2 H, m, C*H2*NH2), 4.14 – 4.22 (2 H, m. NC*H2*), 6.73 (1 H, d, C*H*), 7.65 (1 H, dd, C*H*), 8.22 (1 H, dd, C*H*), 8.48 (1 H, d, C*H*), 8.61 (1 H, dd, C*H*). δ_C (101 MHz, $CDCl_3$) δ 13.90, 20.45, 30.33, 39.97, 40.17, 44.91, 104.37, 110.25, 120.43, 123.05, 124.61, 126.22, 129.75, 131.04, 134.41, 149.66, 164.18, 164.71. *m/z* (ESI) 312.38 ((M+H)$^+$, 100%). All data is consistent with that previously reported in literature.[4]

N-Butyl-4-(2-aminoethyl)-maleimide-1,8-naphthalimide[5] (**1**)

Procedure adapted from literature.[5] **3** (880 mg, 2.7 mmol) and maleic anhydride (4.4 g, 45 mmol) were dissolved in glacial acetic acid (30 mL) and the resulting solution was heated under reflux for 4 h. Acetic acid was removed *in vacuo* before the crude product was purified by flash column chromatography (SiO_2, hexane/EtOAc) to yield **1** as an orange solid (840 mg, 81%). m.p 121-123 °C, δ_H (400 MHz, $CDCl_3$) 0.99 (3 H, t, C*H3*), 1.46 (2 H, m, C*H2*CH3), 1.73 (2 H, m, C*H2*CH2CH3), 3.56 – 3.63 (2 H, m, NHC*H2*), 4.06 – 4.13 (2 H, m, NHCH2C*H2*N), 4.14 – 4.22 (2 H, m. NC*H2*), 6.73 (1 H, d, C*H*), 7.65 (1 H, dd, C*H*), 8.22 (1 H, dd, C*H*), 8.48 (1 H, d, C*H*), 8.61 (1 H, dd, C*H*), 6.10 (1 H, s br, N*H*), 6.66 (1 H, d, C*H*), 6.83 (2 H, s, C*H*C*H*), 7.70 (1 H, dd, C*H*), 8.18 (1 H, dd, C*H*), 8.48 (1 H, d, C*H*), 8.62 (1 H, dd, C*H*). δ_C (101 MHz, $CDCl_3$) 13.89, 20.44, 30.31, 36.88, 40.05, 44.00, 103.90, 111.04, 120.33, 123.11, 125.17, 126.29, 129.65, 131.22, 134.35, 134.50, 149.08, 164.22, 164.70, 171.34. *m/z* (ESI) 392.41 ((M+H)$^+$, 100%).

Poly(acetone oxime acrylate-stat-hydroxyethyl acrylate) (**P1**)

AIBN (1.4 mg, 0.0087 mmol), DDMAT (15.9 mg, 0.044 mmol), **M1** (501 mg, 4.32 mmol) and hydroxyethyl acrylate (HEA) (407 mg, 4.4 mmol) were combined in 1,4-dioxane (2.0 mL) and deoxygenated by argon sparging for 20 min. The solution was then stirred in a preheated oil bath at 70

°C. After 2.6 h, the polymerisation was quenched by rapid cooling in $N_{2(l)}$ and exposure to air. The polymer was purified by dropwise addition into cold Et_2O, redissolved in CH_2Cl_2 and the precipitation repeated twice before drying *in vacuo* to yield **P1** as a yellow-white solid (0.31 g). δ_H (400 MHz, $CDCl_3$) 0.88 (3H, t, CH_3), 1.23 (br, CH_2), 1.5-2.2 (br, polymer backbone), 2.02 (m, C(CH_3)$_2$), 3.65-3.82 (br, OCH_2CH_2OH), 3.97-4.29 (br, OCH_2CH_2OH).

Napthalimide-labelled-poly(acetone oxime acrylate-stat-hydroxyethyl acrylate) (**P2**)
P1 (220 mg, 22 µmol), **3** (85 mg, 220 µmol.), Et_3N (27 µL, 67 µmol), hexylamine (13 µL, 130 µmol) and tris(2-carboxylethyl)phosphine (1.6 mg, 6.5 µmol) were combined in DMF (1.5 mL) and deoxygenated by argon sparging for 20 min. The reaction mixture was then stirred for 18 h. The polymer was purified by dropwise addition into cold Et_2O, redissolved in CH_2Cl_2 and the precipitation repeated twice before drying *in vacuo* to yield **P2** as a yellow solid (200 mg, 90%). δ_H (400 MHz, $CDCl_3$) 1.14 (br s, CH(CH_3)$_2$), 1.29 (m, CH_2), 1.42 – 2.23 (m, polymer backbone), 3.65-3.82 (br, OCH_2CH_2OH), 3.97-4.29 (br, OCH_2CH_2OH), 4.60 (2 H, m, CH_2), 6.90 (1 H, d, C*H*), 8.38 (2 H, m, C*H*C*H*), 8.51 (1 H, d, C*H*).

Napthalimide-labelled-poly(acryloyl hydrazide-stat-hydroxyethyl acrylate) (**P3**)
Hydrazine hydrate (600 µL, 12 mmol) was added to a solution of **P2** (200 mg, 19 µmol) in DMF (1.5 mL) at 0 °C and stirred for 1 h. The solvent was removed in vacuo before the residue was dissolved in water and dialysed against water. The product was isolated by lyophilisation to afford **P3** as a yellow solid (74.4 mg, 44%).

Representative synthesis of functionalised fluorescent copolymers (**P3-carbohydrate**)
P3 (6.1 mg, 0.71 µmol) was dissolved in a solution of aniline (1 mM) and sodium acetate buffer (100 mM, pH 5.5), glucose (6 mg, 35 µmol) was added before the mixture was stirred at 50 °C for 18 h. The reaction mixture was concentrated by centrifugation (2000 *g*, 30 min) (M_w cutoff 3.5 kDa), diluted with water and concentrated twice more (2000 *g*, 30 min) before removing the solvent by lyophilisation to yield **P3-Glc** (6.9 mg, 80%) as a pale yellow solid.

1.2 P1 Copolymerisation kinetics

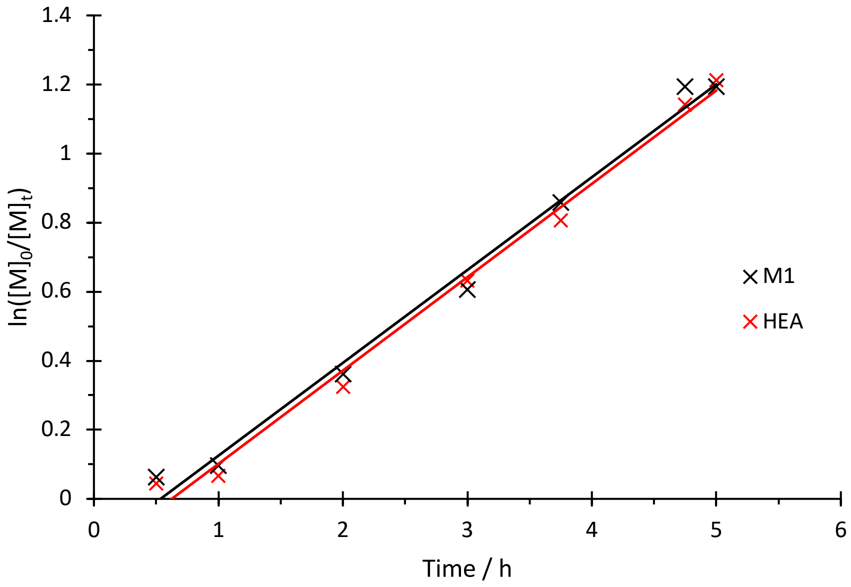

Figure S1 Representative first-order kinetics plots for the copolymerisation of **M1** and hydroxyethyl acrylate (**HEA**).

1.3 Characterisation of P1

Table S1 Characterisation table for polymer **P1**. [a] As determined by conversion analysis using ^{1}H NMR spectroscopy. [b] As determined by gel permeation chromatography in THF (1.0 mL min^{-1}), calibrated against near monodisperse poly(methyl methacrylate) standards.

Polymer	**DDMAT** / eq.	**AIBN** / eq.	**HEA** / eq.	**M1** / eq.	Conversion / %	M_n[a]	M_n[b]	M_w[b]	PDI
**P1**	1	0.2	80	80	49	10300	10100	13500	1.33

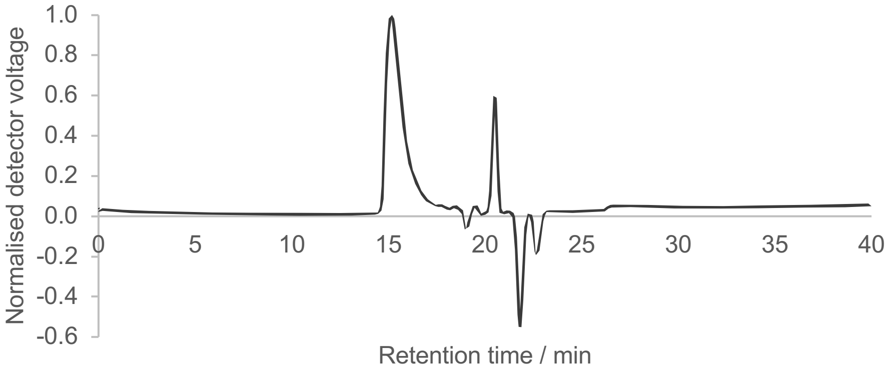

Figure S2 Differential refractive index gel permeation chromatogram of **P1**, acquired using THF (1.0 mL min^{-1}) at 35 °C.

1.4 Characterisation of P3-carbohydrate

Table S2 Table of glycopolymers with associated carbohydrate recognition element, conjugation efficiency and M_n as determined by ^{1}H NMR spectroscopy.

**Polymer**	**Recognition element**	**Attachment / %**	M_n
**P3-Glc**	Glucose	50	12200
**P3-Gal**	Galactose	60	12900
**P3-Man**	Mannose	43	11700
**P3-Fuc**	Fucose	48	11700
**P3-Lac**	Lactose	29	12500
**P3-Mal**	Maltose	25	12000
**P3-GlcNAc**	*N*-Acetylglucosamine	30	11300
**P3-GalNAc**	*N*-Acetylgalactosamine	50	13000
**P3-Neu5Ac**	*N*-Acetylneuraminic acid	25	11700
**P3-Neu5Gc**	*N*-Glycolylneuraminic acid	15	10600

1.5 Emission spectra of P3-carbohydrate

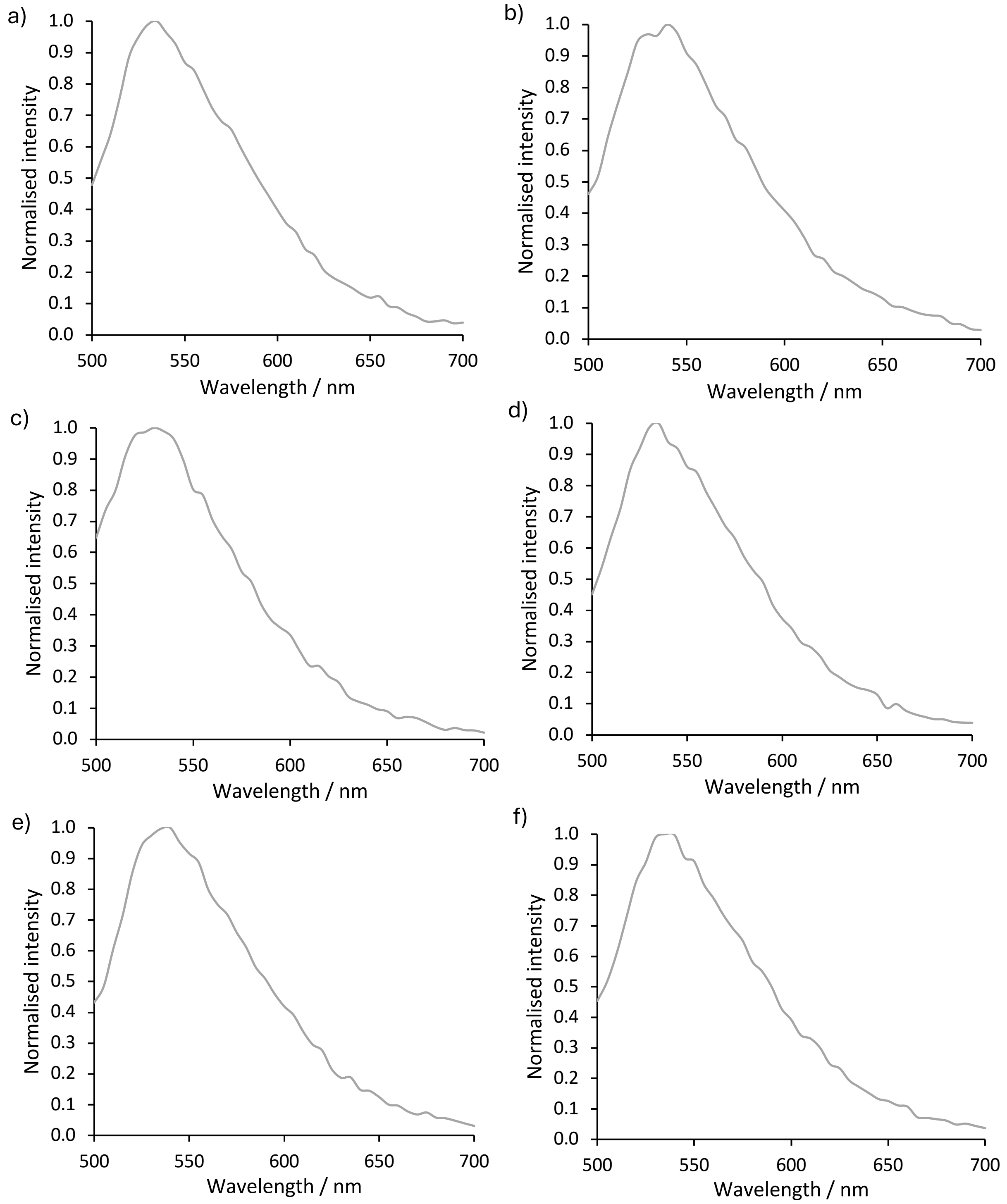

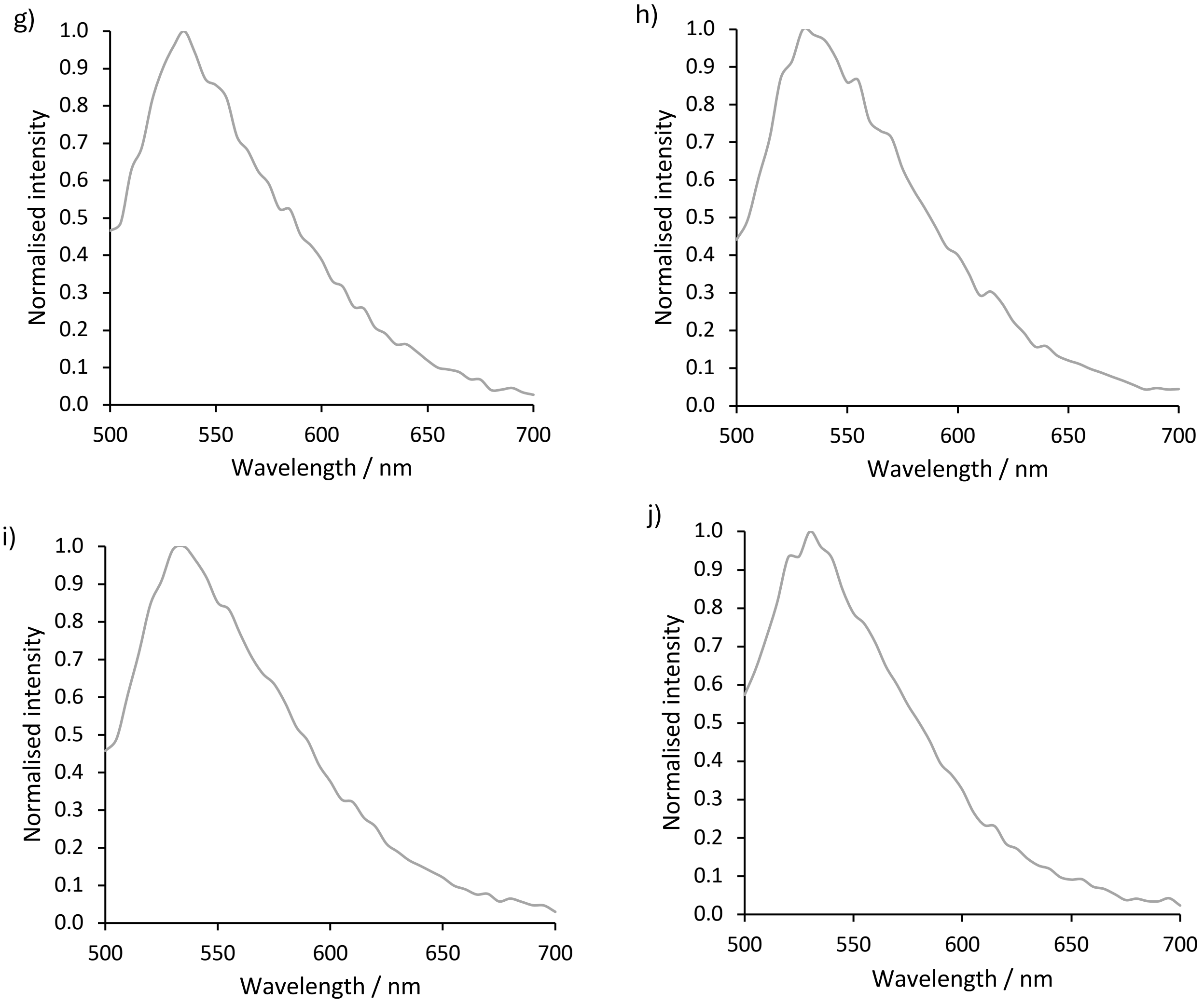

Figure S3 Emission (λ_{ex} = 450 nm) spectra of a) **P3-Glc** b) **P3-Gal** c) **P3-Man** d) **P3-Fuc** e) **P3-Lac** f) **P3-Mal** g) **P3-GlcNAc** h) **P3-GalNAc** i) **P3-Neu5Ac** j) **P3-Neu5Gc**.

2. Discrimination of transposon-insertion mutants

2.1 Method

5.0 µM solutions of each glycopolymer were made up in PBS pH 7.4. Bacteria were grown from glycerol stocks (stored at -80 °C) in Luria−Bertani medium (10 g L^{-1} tryptone, 10 g L^{-1} NaCl, 5 g L^{-1} yeast) at 37 °C for 24 h, maintaining agitation at 180 rpm. Cultures were centrifuged (2500 *g,* 5 min.) to pellet the cells, which were resuspended in sterile PBS pH 7.4 (5 mL). Glycopolymers were pipetted into 96 well plates (Greiner Bio-One) (5.0 µM, 100 µL, 6 replicates). Emission spectra were then obtained by excitation at 450 nm, and measuring emission between 500-700 nm in 5 nm steps. Bacterial suspensions or buffer was then added to each well (10 µL). Solutions were agitated by aspirating and redispersing using the micropipette 3 times, before the emission spectra rerecorded with the same parameters.

Principal component analysis (PCA), linear discriminant analysis (LDA) and hierarchical cluster analysis (HCA) were conducted in IBM SPSS Statistics (version 31.0.0.0 (117)). The principal component method was used to conduct factor analysis to obtain PCA and loading plots. For LDA, samples were grouped by strain with the responses of the 10 glycopolymers as independent variables. HCA was conducted using the "between-groups linkage" cluster method,[6] which defines the distance between two clusters as the average of the pairwise distances between the individuals in one cluster to

individuals in another without standardisation. Variables and cases were clustered to produce dendrograms for glycopolymers and strains respectively.

2.2 Transposon-insertion mutant details

Transposon-insertion mutants were sourced from a collection held at the University of Washington.[7]

P. aeruginosa PAO1	
PAO1 Δ*retS*	PA4856
PAO1 Δ*fimX*	PA4959
PAO1Δ*lecA*	PA2570
PAO1Δ*alg44*	PA3542

2.3 Emission change data

Table S3 Dilution corrected relative changes in fluorescence emission intensity (I/I_0) upon addition of transposon-insertion mutants.

Mutant	Receptor									
	P3-Glc	P3-Gal	P3-Man	P3-Fuc	P3-Mal	P3-Lac	P3-GlcNAc	P3-GalNAc	P3-Neu5Ac	P3-Neu5Gc
Δ*alg44*	1.155	1.161	1.107	1.139	1.159	1.154	1.191	1.150	1.197	1.105
Δ*alg44*	1.131	1.155	1.067	1.163	1.100	1.142	1.247	1.165	1.217	1.105
Δ*alg44*	1.168	1.184	1.077	1.145	1.098	1.144	1.217	1.179	1.211	1.149
Δ*alg44*	1.166	1.159	1.108	1.158	1.137	1.164	1.245	1.181	1.205	1.145
Δ*alg44*	1.159	1.163	1.106	1.153	1.147	1.137	1.221	1.164	1.211	1.169
Δ*alg44*	1.112	1.155	1.106	1.163	1.156	1.135	1.222	1.147	1.198	1.186
PAO1	1.025	0.966	0.946	0.975	0.970	0.952	1.078	0.997	1.019	0.927
PAO1	1.027	0.970	0.949	0.967	0.955	0.938	1.096	0.998	1.004	0.926
PAO1	1.025	0.971	0.952	0.964	0.947	0.934	1.087	1.025	1.010	0.929
PAO1	1.025	0.966	0.959	0.953	0.946	0.926	1.050	1.002	1.024	0.944
PAO1	1.026	0.978	0.962	0.972	0.942	0.926	1.056	0.975	0.963	0.940
PAO1	1.031	0.952	0.957	0.962	0.941	0.931	1.025	0.994	0.995	0.952
Δ*retS*	1.714	1.625	1.240	1.517	1.317	1.579	1.748	1.711	1.229	1.338
Δ*retS*	1.716	1.625	1.353	1.530	1.319	1.583	1.759	1.727	1.211	1.357
Δ*retS*	1.749	1.661	1.385	1.589	1.359	1.592	1.856	1.729	1.234	1.647
Δ*retS*	1.768	1.634	1.431	1.570	1.393	1.615	1.644	1.773	1.230	1.630
Δ*retS*	1.727	1.695	1.421	1.634	1.393	1.659	1.979	1.831	1.251	1.654
Δ*retS*	1.797	1.720	1.421	1.679	1.410	1.678	2.034	1.882	1.274	1.772
Δ*fimX*	1.215	1.209	1.037	1.198	1.160	1.207	1.257	1.217	1.223	1.190
Δ*fimX*	1.218	1.174	1.119	1.198	1.159	1.177	1.252	1.209	1.219	1.158
Δ*fimX*	1.221	1.182	1.119	1.194	1.144	1.156	1.240	1.232	1.228	1.285
Δ*fimX*	1.216	1.185	1.194	1.192	1.168	1.170	1.252	1.185	1.211	1.274
Δ*fimX*	1.208	1.211	1.166	1.223	1.162	1.188	1.280	1.221	1.231	1.271
Δ*fimX*	1.247	1.220	1.173	1.208	1.195	1.206	1.276	1.226	1.253	1.251
Δ*lecA*	1.599	1.500	1.274	1.471	1.305	1.516	1.662	1.643	1.159	1.305
Δ*lecA*	1.594	1.493	1.228	1.500	1.307	1.461	1.563	1.593	1.188	1.232
Δ*lecA*	1.630	1.500	1.193	1.485	1.308	1.487	1.523	1.594	1.196	1.375
Δ*lecA*	1.615	1.558	1.291	1.530	1.322	1.533	1.655	1.629	1.218	1.374

ΔlecA	1.630	1.547	1.277	1.509	1.366	1.521	1.657	1.662	1.211	1.396
ΔlecA	1.608	1.583	1.271	1.625	1.353	1.547	1.695	1.659	1.210	1.445

Table S4 Averages and standard deviations (SD) of dilution corrected relative changes in fluorescence emission intensity (I/I0) upon addition of transposon-insertion mutants.

**Mutant**		**Receptor**									
		**P3-Glc**	**P3-Gal**	**P3-Man**	**P3-Fuc**	**P3-Mal**	**P3-Lac**	**P3-GlcNAc**	**P3-GalNAc**	**P3-Neu5Ac**	**P3-Neu5Gc**
Δalg44	**Mean**	1.149	1.163	1.095	1.154	1.133	1.146	1.224	1.164	1.206	1.143
	**SD**	0.020	0.010	0.017	0.009	0.025	0.010	0.019	0.013	0.007	0.030
**PAO1**	**Mean**	1.027	0.967	0.954	0.965	0.950	0.935	1.065	0.998	1.003	0.937
	**SD**	0.002	0.008	0.006	0.007	0.010	0.009	0.024	0.015	0.020	0.010
ΔretS	**Mean**	1.745	1.660	1.375	1.587	1.365	1.618	1.837	1.776	1.238	1.566
	**SD**	0.030	0.036	0.066	0.056	0.037	0.038	0.136	0.062	0.020	0.161
ΔfimX	**Mean**	1.221	1.197	1.134	1.202	1.165	1.184	1.259	1.215	1.228	1.238
	**SD**	0.012	0.017	0.052	0.010	0.015	0.019	0.014	0.015	0.013	0.047
ΔlecA	**Mean**	1.612	1.530	1.256	1.520	1.327	1.511	1.626	1.630	1.197	1.355
	**SD**	0.014	0.034	0.034	0.051	0.024	0.029	0.061	0.028	0.020	0.069

2.4 LDA canonical discriminant function coefficients

Table S5 Canonical discriminant function coefficients obtained through LDA.

	**Function**			
	**F1**	**F2**	**F3**	**F4**
**P3-Glc**	53.309	-9.321	1.243	50.056
**P3-Gal**	28.953	-6.340	37.552	-65.788
**P3-Man**	2.368	8.144	9.734	1.558
**P3-Fuc**	14.231	-2.199	-42.273	23.783
**P3-Mal**	3.445	0.151	-28.221	-11.113
**P3-Lac**	-8.245	32.386	-0.635	2.665
**P3-GlcNAc**	-2.851	-5.101	5.298	10.122
**P3-GalNAc**	9.537	-18.313	-3.453	-22.269
**P3-Neu5Ac**	-30.110	63.505	10.456	18.385
**P3-Neu5Gc**	-14.632	-2.910	8.946	3.247
**(Constant)**	-79.670	-66.327	0.547	-14.074

2.5 PERMANOVA analysis

Table S6 Full results of pairwise PERMANOVA (adonis2) analysis of the transposon mutant dataset.

**Pairs**	**Df**	**Sums of Squares**	**F Model**	R^2	***P***	***P* adjusted**
Δalg44 vs PAO1	1	0.970538	344.25	0.97	0.003	0.027
Δalg44 vs *ΔretS*	1	6.224056	161.09	0.94	0.005	0.027
Δalg44 vs *ΔfimX*	1	0.078238	13.41	0.57	0.003	0.027
Δalg44 vs *ΔlecA*	1	3.313223	288.15	0.97	0.003	0.027
PAO1 vs *ΔretS*	1	11.54834	305.77	0.97	0.008	0.027

PAO1 vs *ΔfimX*	1	1.540996	310.34	0.97	0.003	0.027
PAO1 vs *ΔlecA*	1	7.375851	693.94	0.99	0.002	0.02
ΔretS vs *ΔfimX*	1	5.035071	123.46	0.93	0.003	0.027
ΔretS vs *ΔlecA*	1	0.534562	11.51	0.54	0.006	0.027
ΔfimX vs *ΔlecA*	1	2.502931	183.44	0.95	0.003	0.027

2.6 Confusion matrices

Table S7 Confusion matrix detailing results of cross-validation of transposon mutant LDA model using 10-glycopolymer array.

		**Predicted group**				
		Δalg44	PAO1	*ΔretS*	*ΔfimX*	*ΔlecA*
**Actual group**	*Δalg44*	5	0	0	1	0
	PAO1	0	6	0	0	0
	ΔretS	0	0	6	0	0
	ΔfimX	1	0	0	5	0
	ΔlecA	0	0	0	0	6

Table S8 Confusion matrix detailing results of cross-validation of transposon mutant LDA model using reduced array consisting of **P3-Glc**, **P3-Neu5Gc**, **P3-GlcNAc** and **P3-Neu5Ac**.

		**Predicted group**				
		Δalg44	PAO1	*ΔretS*	*ΔfimX*	*ΔlecA*
**Actual group**	*Δalg44*	6	0	0	0	0
	PAO1	0	6	0	0	0
	ΔretS	0	0	6	0	0
	ΔfimX	0	0	0	6	0
	ΔlecA	0	0	0	0	6

2.7 Hold-out validation

Hold-out validation was conducted on the dataset using 4 datapoints for each strain (Table S9, green) to construct an LDA scoring model which was used to classify the remaining 2 datapoints for each strain (Table S9; red). The hold-out dataset was classified with 100% accuracy with the full array (Table S10; 10/10) and reduced array (Table S11; 10/10).

Table S9 Dilution corrected relative changes in fluorescence emission intensity (I/I_0) upon addition of transposon mutants, with data used for training model highlighted in green and the hold-out set in red.

**Mutant**	**Receptor**									
	**P3-Glc**	**P3-Gal**	**P3-Man**	**P3-Fuc**	**P3-Mal**	**P3-Lac**	**P3-GlcNAc**	**P3-GalNAc**	**P3-Neu5Ac**	**P3-Neu5Gc**
Δalg44	1.155	1.161	1.107	1.139	1.159	1.154	1.191	1.150	1.197	1.105
Δalg44	1.131	1.155	1.067	1.163	1.100	1.142	1.247	1.165	1.217	1.105
Δalg44	1.168	1.184	1.077	1.145	1.098	1.144	1.217	1.179	1.211	1.149
Δalg44	1.166	1.159	1.108	1.158	1.137	1.164	1.245	1.181	1.205	1.145
Δalg44	1.159	1.163	1.106	1.153	1.147	1.137	1.221	1.164	1.211	1.169
Δalg44	1.112	1.155	1.106	1.163	1.156	1.135	1.222	1.147	1.198	1.186
**PAO1**	1.025	0.966	0.946	0.975	0.970	0.952	1.078	0.997	1.019	0.927

**PAO1**	1.027	0.970	0.949	0.967	0.955	0.938	1.096	0.998	1.004	0.926
**PAO1**	1.025	0.971	0.952	0.964	0.947	0.934	1.087	1.025	1.010	0.929
**PAO1**	1.025	0.966	0.959	0.953	0.946	0.926	1.050	1.002	1.024	0.944
**PAO1**	1.026	0.978	0.962	0.972	0.942	0.926	1.056	0.975	0.963	0.940
**PAO1**	1.031	0.952	0.957	0.962	0.941	0.931	1.025	0.994	0.995	0.952
**Δ*retS***	1.714	1.625	1.240	1.517	1.317	1.579	1.748	1.711	1.229	1.338
**Δ*retS***	1.716	1.625	1.353	1.530	1.319	1.583	1.759	1.727	1.211	1.357
**Δ*retS***	1.749	1.661	1.385	1.589	1.359	1.592	1.856	1.729	1.234	1.647
**Δ*retS***	1.768	1.634	1.431	1.570	1.393	1.615	1.644	1.773	1.230	1.630
**Δ*retS***	1.727	1.695	1.421	1.634	1.393	1.659	1.979	1.831	1.251	1.654
**Δ*retS***	1.797	1.720	1.421	1.679	1.410	1.678	2.034	1.882	1.274	1.772
**Δ*fimX***	1.215	1.209	1.037	1.198	1.160	1.207	1.257	1.217	1.223	1.190
**Δ*fimX***	1.218	1.174	1.119	1.198	1.159	1.177	1.252	1.209	1.219	1.158
**Δ*fimX***	1.221	1.182	1.119	1.194	1.144	1.156	1.240	1.232	1.228	1.285
**Δ*fimX***	1.216	1.185	1.194	1.192	1.168	1.170	1.252	1.185	1.211	1.274
**Δ*fimX***	1.208	1.211	1.166	1.223	1.162	1.188	1.280	1.221	1.231	1.271
**Δ*fimX***	1.247	1.220	1.173	1.208	1.195	1.206	1.276	1.226	1.253	1.251
**Δ*lecA***	1.599	1.500	1.274	1.471	1.305	1.516	1.662	1.643	1.159	1.305
**Δ*lecA***	1.594	1.493	1.228	1.500	1.307	1.461	1.563	1.593	1.188	1.232
**Δ*lecA***	1.630	1.500	1.193	1.485	1.308	1.487	1.523	1.594	1.196	1.375
**Δ*lecA***	1.615	1.558	1.291	1.530	1.322	1.533	1.655	1.629	1.218	1.374
**Δ*lecA***	1.630	1.547	1.277	1.509	1.366	1.521	1.657	1.662	1.211	1.396
**Δ*lecA***	1.608	1.583	1.271	1.625	1.353	1.547	1.695	1.659	1.210	1.445

Table S10 Confusion matrix detailing results of hold-out validation of transposon mutant dataset.

		**Predicted group**				
		Δalg44	PAO1	*ΔretS*	*ΔfimX*	*ΔlecA*
**Actual group**	*Δalg44*	2	0	0	0	0
	PAO1	0	2	0	0	0
	ΔretS	0	0	2	0	0
	ΔfimX	0	0	0	2	0
	ΔlecA	0	0	0	0	2

Table S11 Confusion matrix detailing results of hold-out validation of transposon mutant dataset model using reduced array consisting of **P3-Glc**, **P3-Neu5Gc**, **P3-GlcNAc** and **P3-Neu5Ac**.

		**Predicted group**				
		Δalg44	PAO1	*ΔretS*	*ΔfimX*	*ΔlecA*
**Actual group**	*Δalg44*	2	0	0	0	0
	PAO1	0	2	0	0	0
	ΔretS	0	0	2	0	0
	ΔfimX	0	0	0	2	0
	ΔlecA	0	0	0	0	2

2.8 Principal component analysis (PCA)

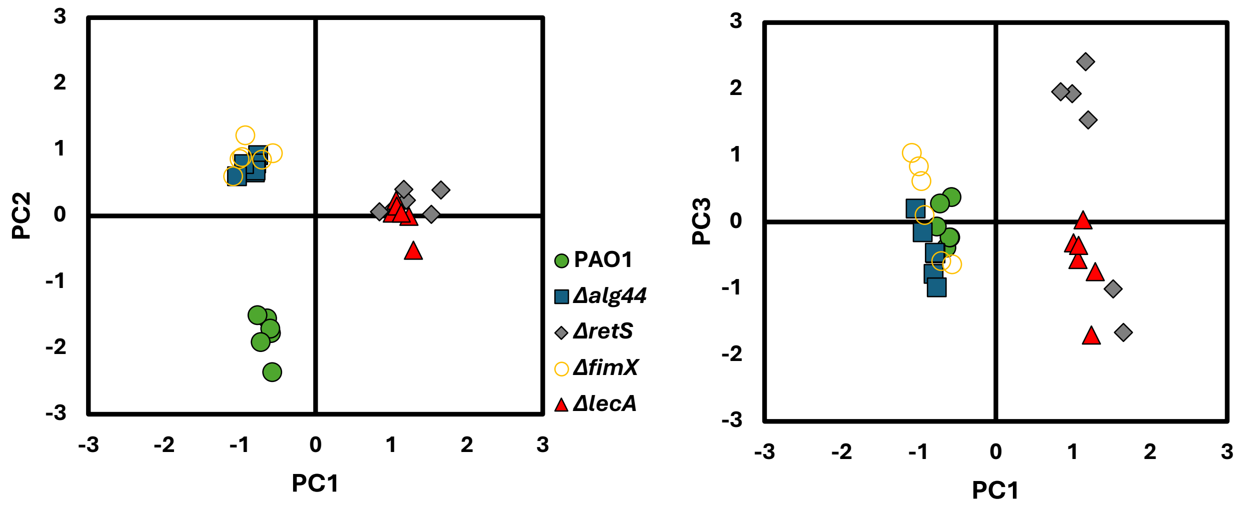

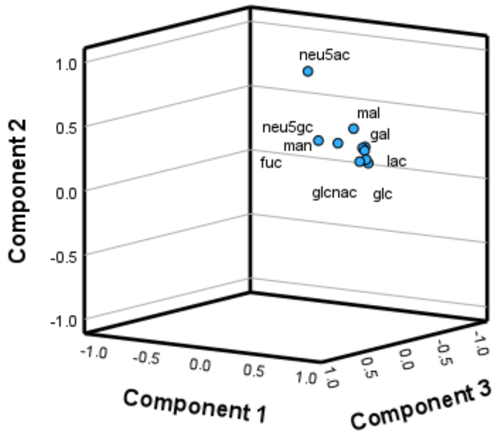

Figure S4 PCA loading plots visualising relationship between glycopolymers and principal components for transposon-insertion mutant dataset.

2.9 LDA including PAO1 dilutions

To assess the response of the sensor array to analytes at different effective concentrations, PAO1 was grown to saturation from a glycerol stock (stored at -80 °C) in Luria–Bertani medium (10 g L^{-1} tryptone, 10 g L^{-1} NaCl, 5 g L^{-1} yeast) at 37 °C for 24 h, maintaining agitation at 180 rpm. The culture was centrifuged (2500 *g,* 5 min.) to pellet the cells, which were resuspended in sterile PBS pH 7.4 (5 mL). Aliquots of this suspension (1 mL) were then diluted with PBS pH 7.4 (1 mL or 3 mL) to achieve 1 in 2 and 1 in 4 dilutions respectively. PAO1 dilutions were included as ungrouped cases and assigned by the LDA model in SPSS. Dilutions were scored as PAO1 in all cases (Table S13; 12/12).

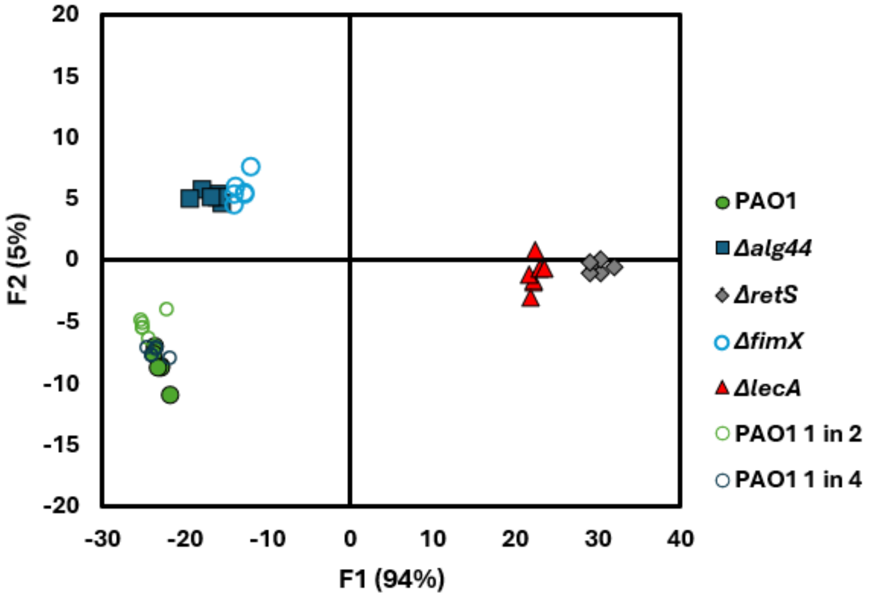

Figure S5 Canonical LDA score plots for functions 1 & 2 for the analysis of the transposon mutants and dilutions of PAO1 performed in sextuplicate (5.0 µM receptors, pH 7.4).

Table S12 Dilution corrected data for PAO1 dilutions in PBS

Dilution	Receptor									
	P3-Glc	P3-Gal	P3-Man	P3-Fuc	P3-Mal	P3-Lac	P3-GlcNAc	P3-GalNAc	P3-Neu5Ac	P3-Neu5Gc
**1 in 2**	0.994	0.969	0.978	0.973	0.991	0.981	1.041	0.991	1.021	0.948
**1 in 2**	0.990	0.978	0.988	0.987	0.991	0.976	1.053	0.987	1.026	0.953
**1 in 2**	0.992	0.984	0.991	0.979	0.990	0.981	1.039	0.986	1.020	0.963
**1 in 2**	0.998	0.991	1.000	0.956	0.990	0.986	1.019	0.994	1.003	0.967
**1 in 2**	0.998	0.992	1.000	0.989	0.989	0.977	0.989	0.991	1.012	0.972
**1 in 2**	0.984	0.989	1.000	0.991	0.993	0.974	1.006	0.983	1.019	0.976
**1 in 4**	1.003	0.979	0.985	0.983	0.991	0.978	1.012	0.986	0.996	0.951
**1 in 4**	0.989	0.982	0.988	0.986	0.992	0.979	1.061	0.981	0.996	0.959
**1 in 4**	0.991	0.987	0.987	0.982	0.990	0.972	1.043	0.981	0.988	0.964
**1 in 4**	0.986	0.981	0.996	0.989	0.986	0.973	1.001	0.984	0.984	0.973
**1 in 4**	0.988	0.992	0.993	0.987	0.989	0.972	0.967	0.977	0.982	0.973
**1 in 4**	0.995	0.982	0.995	0.989	0.988	0.969	0.975	0.976	0.988	0.979

Table S13 Confusion matrix detailing results of LDA after including dilutions of PAO1 as ungrouped cases

		Predicted group				
		Δalg44	PAO1	*ΔretS*	*ΔfimX*	*ΔlecA*
**Actual group**	*Δalg44*	6	0	0	0	0
	PAO1	0	6	0	0	0
	ΔretS	0	0	6	0	0
	ΔfimX	0	0	0	6	0
	ΔlecA	0	0	0	0	6
	1 in 2	0	6	0	0	0
	1 in 4	0	6	0	0	0

3. Discrimination of clinical isolates

3.1 Method

5.0 µM solutions of each glycopolymer were made up in PBS pH 7.4. Bacteria were grown from glycerol stocks (stored at -80 °C) in Luria−Bertani medium (10 g L^{-1} tryptone, 10 g L^{-1} NaCl, 5 g L^{-1} yeast) at 37 °C for 24 h, maintaining agitation at 180 rpm. Cultures were centrifuged (2500 *g,* 5 min.) to pellet the cells, which were resuspended in sterile PBS pH 7.4 (5 mL). Glycopolymers were pipetted into 96 well plates (Greiner Bio-One) (5.0 µM, 100 µL, 6 replicates). Emission spectra were then obtained by excitation at 450 nm, and measuring emission between 500-700 nm in 5 nm steps. Bacterial suspensions or buffer was then added to each well (10 µL). Solutions were agitated by aspirating and redispersing using the micropipette 3 times, before the emission spectra rerecorded with the same parameters.

Principal component analysis (PCA), linear discriminant analysis (LDA) and hierarchical cluster analysis (HCA) were conducted in IBM SPSS Statistics (version 31.0.0.0 (117)). The principle component method was used to conduct factor analysis to obtain PCA and loading plots. For LDA, samples were grouped by strain with the responses of the 10 glycopolymers as independent variables. HCA was conducted using the "between-groups linkage" cluster method without standardisation. Variables and cases were clustered to produce dendrograms for glycopolymers and strains respectively.

3.2 Clinical isolate details.

Table S14 Strain number, patient ID and the age of the infection corresponding to the genotypes of the clinical isolates used to test our array.

Genotype	Isolate name	Patient	Infection age of genotype / years
**DK32**	Pa247	CF382	5.5
**DK15**	Pa427	CF236	0
**DK09**	Pa80	CF408	1.8
**DK28**	Pa202	CF496	0
**DK29**	Pa209	CF496	3.5

3.3 Emission change data

Table S15 Dilution corrected relative changes in fluorescence emission intensity upon addition of clinical isolates.

Isolate	Receptor									
	P3-Glc	P3-Gal	P3-Man	P3-Fuc	P3-Mal	P3-Lac	P3-GlcNAc	P3-GalNAc	P3-Neu5Ac	P3-Neu5Gc
**PAO1**	1.025	0.966	0.946	0.975	0.970	0.952	1.078	0.997	1.019	0.927
**PAO1**	1.027	0.970	0.949	0.967	0.955	0.938	1.096	0.998	1.004	0.926
**PAO1**	1.025	0.971	0.952	0.964	0.947	0.934	1.087	1.025	1.010	0.929
**PAO1**	1.025	0.966	0.959	0.953	0.946	0.926	1.050	1.002	1.024	0.944
**PAO1**	1.026	0.978	0.962	0.972	0.942	0.926	1.056	0.975	0.963	0.940
**PAO1**	1.031	0.952	0.957	0.962	0.941	0.931	1.025	0.994	0.995	0.952
**DK32**	1.159	1.254	1.109	1.243	1.197	1.180	1.221	1.253	1.136	1.027
**DK32**	1.205	1.258	1.074	1.209	1.172	1.227	1.237	1.265	1.112	1.040
**DK32**	1.197	1.257	1.073	1.220	1.200	1.213	1.266	1.268	1.153	1.072
**DK32**	1.216	1.265	0.988	1.238	1.218	1.237	1.287	1.270	1.152	1.067

**DK32**	1.235	1.299	1.058	1.244	1.202	1.216	1.305	1.314	1.187	1.056
**DK32**	1.241	1.375	1.024	1.294	1.257	1.318	1.348	1.351	1.187	1.122
**DK15**	1.406	1.506	1.305	1.491	1.310	1.514	1.671	1.614	1.302	1.341
**DK15**	1.584	1.513	1.296	1.497	1.293	1.534	1.648	1.616	1.289	1.270
**DK15**	1.601	1.620	1.318	1.530	1.356	1.559	1.710	1.638	1.311	1.432
**DK15**	1.619	1.606	1.337	1.563	1.376	1.590	1.677	1.645	1.285	1.382
**DK15**	1.631	1.649	1.290	1.577	1.419	1.611	1.744	1.755	1.313	1.480
**DK15**	1.562	1.682	1.423	1.650	1.443	1.628	1.874	1.789	1.322	1.558
**DK09**	1.112	1.138	1.134	1.139	1.124	1.184	1.163	1.133	1.128	1.146
**DK09**	1.120	1.154	1.098	1.129	1.019	1.128	1.129	1.125	1.131	1.193
**DK09**	1.145	1.143	1.090	1.139	1.141	1.150	1.184	1.155	1.133	1.161
**DK09**	1.145	1.163	1.106	1.139	1.118	1.127	1.215	1.163	1.132	1.148
**DK09**	1.150	1.172	1.089	1.164	1.058	1.153	1.215	1.146	1.152	1.153
**DK09**	1.154	1.154	1.096	1.158	1.036	1.149	1.226	1.145	1.142	1.271
**DK28**	1.221	1.308	0.916	1.304	1.237	1.364	1.406	1.450	1.143	0.974
**DK28**	1.265	1.313	0.814	1.259	1.212	1.362	1.423	1.419	1.157	1.020
**DK28**	1.249	1.305	0.840	1.292	1.244	1.327	1.442	1.427	1.144	1.025
**DK28**	1.197	1.224	0.814	1.280	1.234	1.362	1.460	1.439	1.167	0.895
**DK28**	1.208	1.330	0.829	1.286	1.271	1.330	1.512	1.443	1.176	1.014
**DK29**	1.103	1.090	0.975	1.106	1.040	1.108	1.140	1.619	1.105	0.875
**DK29**	1.082	1.080	0.877	1.083	1.082	1.148	1.135	1.093	1.127	0.963
**DK29**	1.145	1.094	0.888	1.100	1.106	1.107	1.162	1.762	1.135	0.976
**DK29**	1.097	1.071	0.796	1.100	1.123	1.096	1.261	1.086	1.134	0.964
**DK29**	1.197	1.086	0.875	1.099	1.095	1.119	1.170	1.132	1.162	0.942

Table S16 Averages and standard deviations (SD) of dilution corrected relative changes in fluorescence emission intensity (I/I0) upon addition of clinical isolates.

**Isolate**		**Receptor**									
		**P3-Glc**	**P3-Gal**	**P3-Man**	**P3-Fuc**	**P3-Mal**	**P3-Lac**	**P3-GlcNAc**	**P3-GalNAc**	**P3-Neu5Ac**	**P3-Neu5Gc**
**PAO1**	**Mean**	1.027	0.967	0.954	0.965	0.950	0.935	1.065	0.998	1.003	0.937
	**SD**	0.002	0.008	0.006	0.007	0.010	0.009	0.024	0.015	0.020	0.010
**DK32**	**Mean**	1.209	1.285	1.054	1.241	1.208	1.232	1.277	1.287	1.154	1.064
	**SD**	0.027	0.043	0.039	0.027	0.026	0.042	0.042	0.034	0.027	0.030
**DK15**	**Mean**	1.567	1.596	1.328	1.551	1.366	1.572	1.721	1.676	1.304	1.410
	**SD**	0.075	0.066	0.045	0.054	0.054	0.041	0.075	0.070	0.013	0.093
**DK09**	**Mean**	1.138	1.154	1.102	1.145	1.083	1.149	1.188	1.144	1.136	1.179
	**SD**	0.016	0.011	0.015	0.012	0.047	0.019	0.034	0.013	0.008	0.044
**DK28**	**Mean**	1.228	1.296	0.843	1.284	1.240	1.349	1.449	1.436	1.157	0.986
	**SD**	0.025	0.037	0.038	0.015	0.019	0.017	0.036	0.011	0.013	0.049
**DK29**	**Mean**	1.125	1.084	0.882	1.098	1.089	1.115	1.174	1.338	1.133	0.944
	**SD**	0.042	0.008	0.057	0.008	0.028	0.018	0.046	0.291	0.018	0.036

3.4 LDA canonical discriminant function coefficient

Table S17 Canonical discriminant function coefficients for receptors obtained through LDA.

	Function				
	**F1**	**F2**	**F3**	**F4**	**F5**
**P3-Glc**	8.390	12.802	12.140	3.066	16.264
**P3-Gal**	-6.185	-22.604	-4.530	-38.159	-3.348
**P3-Man**	13.183	29.169	5.934	-7.929	3.254
**P3-Fuc**	8.023	-32.605	-31.462	0.172	1.465
**P3-Mal**	-6.920	0.582	-14.537	-8.086	7.282
**P3-Lac**	19.308	-2.123	12.870	20.687	-24.997
**P3-GlcNAc**	6.196	7.041	36.244	1.628	2.354
**P3-GalNAc**	-0.894	-1.998	0.337	4.544	2.632
**P3-Neu5Ac**	22.177	12.450	-45.674	19.772	19.818
**P3-Neu5Gc**	-5.223	19.038	-4.274	13.353	-10.477
**(Constant)**	-68.379	-18.160	33.017	-10.066	-16.455

3.5 PERMANOVA analysis

Table S18 Full results of pairwise PERMANOVA (adonis2) analysis of the clinical isolates dataset.

**Pairs**	**Df**	**Sums Of Squares**	**F Model**	**R^2**	***p***	***p* adjusted**
PAO1 vs DK32	1	1.627096	200.68	0.95	0.004	0.03
PAO1 vs DK15	1	8.866111	364.7	0.97	0.002	0.028
PAO1 vs DK09	1	0.833357	167.35	0.94	0.003	0.03
PAO1 vs DK28	1	2.407373	421.86	0.98	0.002	0.028
PAO1 vs DK29	1	0.660969	12.29	0.58	0.003	0.03
DK32 vs DK15	1	3.08443	101.23	0.91	0.006	0.03
DK32 vs DK09	1	0.293818	26.38	0.73	0.001	0.015
DK32 vs DK28	1	0.325993	25.98	0.74	0.005	0.03
DK32 vs DK29	1	0.418313	6.9	0.43	0.004	0.03
DK15 vs DK09	1	4.512055	165.03	0.94	0.004	0.03
DK15 vs DK28	1	2.487386	81.42	0.9	0.002	0.028
DK15 vs DK29	1	4.931625	62.72	0.87	0.003	0.03
DK09 vs DK28	1	1.009145	111.23	0.93	0.003	0.03
DK09 vs DK29	1	0.408191	7.14	0.44	0.002	0.028
DK28 vs DK29	1	0.641028	9.94	0.55	0.007	0.03

3.6 Confusion matrices

Table S19 Confusion matrix detailing results of cross-validation of clinical isolates LDA model using 10-glycopolymer array.

		Predicted group					
		PAO1	DK32	DK15	DK09	DK28	DK29
**Actual group**	PAO1	6	0	0	0	0	0
	DK32	0	6	0	0	0	0
	DK15	0	0	6	0	0	0
	DK09	0	0	0	6	0	0
	DK28	0	0	0	0	5	0
	DK29	0	0	0	0	0	5

Table S20 Confusion matrix detailing results of cross-validation of clinical isolates LDA model reduced glycopolymer array consisting of **P3-Lac**, **P3-Mal**, **P3-GalNAc** and **P3-Neu5Gc**.

		Predicted group					
		PAO1	DK32	DK15	DK09	DK28	DK29
**Actual group**	PAO1	6	0	0	0	0	0
	DK32	0	5	0	0	1	0
	DK15	0	0	6	0	0	0
	DK09	0	0	0	6	0	0
	DK28	0	0	0	0	5	0
	DK29	0	0	0	0	0	5

3.7 Hold-out validation

Hold-out validation was conducted on the dataset using 4 datapoints for each isolate (Table S21, green) to construct an LDA scoring model which was used to classify the remaining 2 datapoints for each strain (Table S20, red). The hold-out dataset was classified with 90% accuracy for the full array (Table S22; 9/10) and reduced array (Table S23; 9/10), both corresponding to a single misclassification of DK32 as DK28.

Table S21 Dilution corrected relative changes in fluorescence emission intensity (I/I_0) upon addition of clinical isolates, with data used for training model highlighted in green and the hold-out set in red.

**Isolate**	**Receptor**									
	**P3-Glc**	**P3-Gal**	**P3-Man**	**P3-Fuc**	**P3-Mal**	**P3-Lac**	**P3-GlcNAc**	**P3-GalNAc**	**P3-Neu5Ac**	**P3-Neu5Gc**
**PAO1**	1.025	0.966	0.946	0.975	0.970	0.952	1.078	0.997	1.019	0.927
**PAO1**	1.027	0.970	0.949	0.967	0.955	0.938	1.096	0.998	1.004	0.926
**PAO1**	1.025	0.971	0.952	0.964	0.947	0.934	1.087	1.025	1.010	0.929
**PAO1**	1.025	0.966	0.959	0.953	0.946	0.926	1.050	1.002	1.024	0.944

**PAO1**	1.026	0.978	0.962	0.972	0.942	0.926	1.056	0.975	0.963	0.940
**PAO1**	1.031	0.952	0.957	0.962	0.941	0.931	1.025	0.994	0.995	0.952
**DK32**	1.159	1.254	1.109	1.243	1.197	1.180	1.221	1.253	1.136	1.027
**DK32**	1.205	1.258	1.074	1.209	1.172	1.227	1.237	1.265	1.112	1.040
**DK32**	1.197	1.257	1.073	1.220	1.200	1.213	1.266	1.268	1.153	1.072
**DK32**	1.216	1.265	0.988	1.238	1.218	1.237	1.287	1.270	1.152	1.067
**DK32**	1.235	1.299	1.058	1.244	1.202	1.216	1.305	1.314	1.187	1.056
**DK32**	1.241	1.375	1.024	1.294	1.257	1.318	1.348	1.351	1.187	1.122
**DK15**	1.406	1.506	1.305	1.491	1.310	1.514	1.671	1.614	1.302	1.341
**DK15**	1.584	1.513	1.296	1.497	1.293	1.534	1.648	1.616	1.289	1.270
**DK15**	1.601	1.620	1.318	1.530	1.356	1.559	1.710	1.638	1.311	1.432
**DK15**	1.619	1.606	1.337	1.563	1.376	1.590	1.677	1.645	1.285	1.382
**DK15**	1.631	1.649	1.290	1.577	1.419	1.611	1.744	1.755	1.313	1.480
**DK15**	1.562	1.682	1.423	1.650	1.443	1.628	1.874	1.789	1.322	1.558
**DK09**	1.112	1.138	1.134	1.139	1.124	1.184	1.163	1.133	1.128	1.146
**DK09**	1.120	1.154	1.098	1.129	1.019	1.128	1.129	1.125	1.131	1.193
**DK09**	1.145	1.143	1.090	1.139	1.141	1.150	1.184	1.155	1.133	1.161
**DK09**	1.145	1.163	1.106	1.139	1.118	1.127	1.215	1.163	1.132	1.148
**DK09**	1.150	1.172	1.089	1.164	1.058	1.153	1.215	1.146	1.152	1.153
**DK09**	1.154	1.154	1.096	1.158	1.036	1.149	1.226	1.145	1.142	1.271
**DK28**	1.221	1.308	0.916	1.304	1.237	1.364	1.406	1.450	1.143	0.974
**DK28**	1.265	1.313	0.814	1.259	1.212	1.362	1.423	1.419	1.157	1.020
**DK28**	1.249	1.305	0.840	1.292	1.244	1.327	1.442	1.427	1.144	1.025
**DK28**	1.197	1.224	0.814	1.280	1.234	1.362	1.460	1.439	1.167	0.895
**DK28**	1.208	1.330	0.829	1.286	1.271	1.330	1.512	1.443	1.176	1.014
**DK29**	1.103	1.090	0.975	1.106	1.040	1.108	1.140	1.619	1.105	0.875
**DK29**	1.082	1.080	0.877	1.083	1.082	1.148	1.135	1.093	1.127	0.963
**DK29**	1.145	1.094	0.888	1.100	1.106	1.107	1.162	1.762	1.135	0.976
**DK29**	1.097	1.071	0.796	1.100	1.123	1.096	1.261	1.086	1.134	0.964
**DK29**	1.197	1.086	0.875	1.099	1.095	1.119	1.170	1.132	1.162	0.942

Table S22 Confusion matrix detailing results of hold-out validation of clinical isolate dataset.

		Predicted group					
		PAO1	DK32	DK15	DK09	DK28	DK29
Actual group	PAO1	2	0	0	0	0	0
	DK32	0	1	0	0	1	0
	DK15	0	0	2	0	0	0
	DK09	0	0	0	2	0	0
	DK28	0	0	0	0	1	0
	DK29	0	0	0	0	0	1

Table S23 Confusion matrix detailing results of hold-out validation of clinical isolate dataset reduced glycopolymer array consisting of **P3-Lac**, **P3-Mal**, **P3-GalNAc** and **P3-Neu5Gc**.

		Predicted group					
		PAO1	DK32	DK15	DK09	DK28	DK29
Actual group	PAO1	2	0	0	0	0	0
	DK32	0	1	0	0	1	0
	DK15	0	0	2	0	0	0
	DK09	0	0	0	2	0	0
	DK28	0	0	0	0	1	0
	DK29	0	0	0	0	0	1

3.8 Principal component analysis (PCA) results

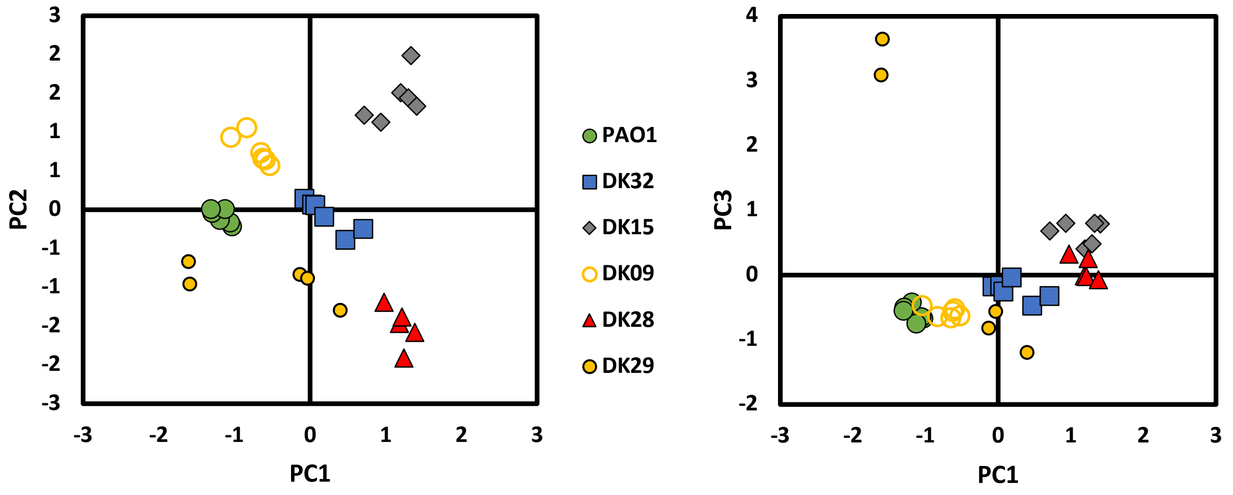

Figure S6 PCA score plots for functions 1 & 2 and functions 1 & 3 for the analysis of the clinical isolates (5.0 µM receptors, pH 7.4).

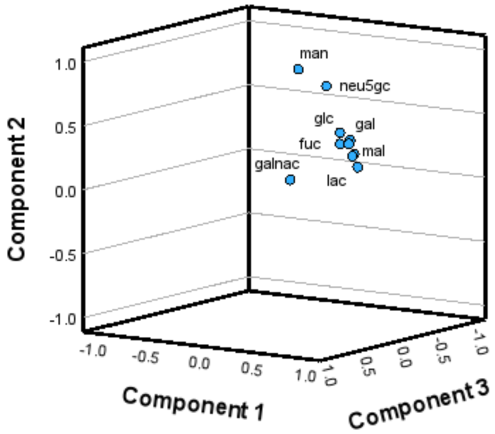

Figure S7 PCA loading plot visualising relationship between glycopolymers and principal components for clinical isolate dataset.

3.9 LDA including PAO1 dilutions

To assess the response of the sensor array to analytes at different effective concentrations, PAO1 was grown to saturation from a glycerol stock (stored at -80 °C) in Luria–Bertani medium (10 g L^{-1} tryptone, 10 g L^{-1} NaCl, 5 g L^{-1} yeast) at 37 °C for 24 h, maintaining agitation at 180 rpm. The culture was centrifuged (2500 *g*, 5 min.) to pellet the cells, which were resuspended in sterile PBS pH 7.4 (5 mL). Aliquots of this suspension (1 mL) were then diluted with PBS pH 7.4 (1 mL or 3 mL) to achieve 1 in 2 and 1 in 4 dilutions respectively. PAO1 dilutions were included as ungrouped cases and assigned by the LDA model in SPSS. Dilutions were scored as PAO1 in all cases (Table S25; 12/12).

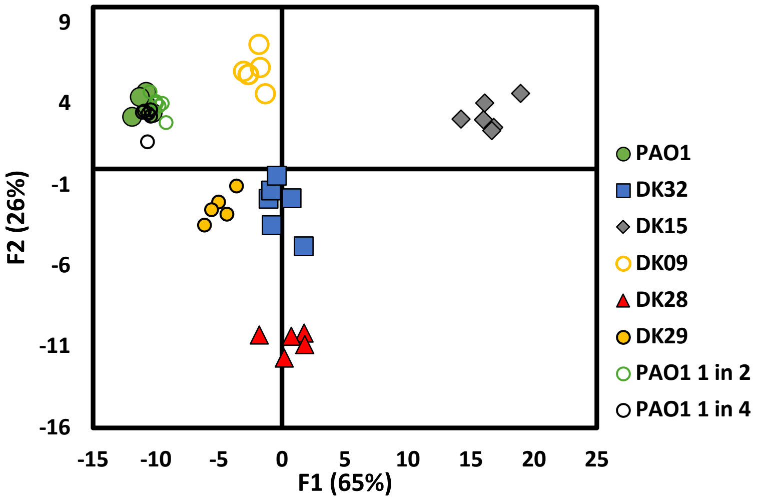

Figure S8 Canonical LDA score plots for functions 1 & 2 for the analysis of the clinical isolates and dilutions of PAO1 performed (5.0 µM receptors, pH 7.4).

Table S24 Dilution corrected data for PAO1 dilutions in PBS

Dilution	Receptor									
	**P3-Glc**	**P3-Gal**	**P3-Man**	**P3-Fuc**	**P3-Mal**	**P3-Lac**	**P3-GlcNAc**	**P3-GalNAc**	**P3-Neu5Ac**	**P3-Neu5Gc**
**1 in 2**	0.994	0.969	0.978	0.973	0.991	0.981	1.041	0.991	1.021	0.948
**1 in 2**	0.990	0.978	0.988	0.987	0.991	0.976	1.053	0.987	1.026	0.953
**1 in 2**	0.992	0.984	0.991	0.979	0.990	0.981	1.039	0.986	1.020	0.963
**1 in 2**	0.998	0.991	1.000	0.956	0.990	0.986	1.019	0.994	1.003	0.967
**1 in 2**	0.998	0.992	1.000	0.989	0.989	0.977	0.989	0.991	1.012	0.972
**1 in 2**	0.984	0.989	1.000	0.991	0.993	0.974	1.006	0.983	1.019	0.976
**1 in 4**	1.003	0.979	0.985	0.983	0.991	0.978	1.012	0.986	0.996	0.951
**1 in 4**	0.989	0.982	0.988	0.986	0.992	0.979	1.061	0.981	0.996	0.959
**1 in 4**	0.991	0.987	0.987	0.982	0.990	0.972	1.043	0.981	0.988	0.964
**1 in 4**	0.986	0.981	0.996	0.989	0.986	0.973	1.001	0.984	0.984	0.973
**1 in 4**	0.988	0.992	0.993	0.987	0.989	0.972	0.967	0.977	0.982	0.973
**1 in 4**	0.995	0.982	0.995	0.989	0.988	0.969	0.975	0.976	0.988	0.979

Table S25 Confusion matrix detailing results of LDA after including dilutions of PAO1 as ungrouped cases.

		Predicted group					
		PAO1	DK32	DK15	DK09	DK28	DK29
**Actual group**	PAO1	6	0	0	0	0	0
	DK32	0	6	0	0	0	0
	DK15	0	0	6	0	0	0
	DK09	0	0	0	6	0	0
	DK28	0	0	0	0	5	0
	DK29	0	0	0	0	0	5
	1 in 2	6	0	0	0	0	0
	1 in 4	6	0	0	0	0	0

4. Discrimination of lung pathogens

4.1 Method

5.0 µM solutions of each glycopolymer were made up in PBS pH 7.4. Bacteria were grown from glycerol stocks (stored at -80 °C) in Luria−Bertani medium (10 g L^{-1} tryptone, 10 g L^{-1} NaCl, 5 g L^{-1} yeast) at 37 °C for 24 h, maintaining agitation at 180 rpm. Cultures were centrifuged (2500 *g,* 5 min.) to pellet the cells, which were resuspended in sterile PBS pH 7.4 (5 mL). Glycopolymers were pipetted into 96 well plates (Greiner Bio-One) (5.0 µM, 100 µL, 6 replicates). Emission spectra were then obtained by excitation at 450 nm, and measuring emission between 500-700 nm in 5 nm steps. Bacterial suspensions or buffer was then added to each well (10 µL). Solutions were agitated by aspirating and redispersing using the micropipette 3 times, before the emission spectra rerecorded with the same parameters.

Principal component analysis (PCA), linear discriminant analysis (LDA) and hierarchical cluster analysis (HCA) were conducted in IBM SPSS Statistics (version 31.0.0.0 (117)). The principal component method was used to conduct factor analysis to obtain PCA and loading plots. For LDA, samples were grouped by strain with the responses of the 10 glycopolymers as independent variables. HCA was conducted using the "between-groups linkage" cluster method without standardisation. Variables and cases were clustered to produce dendrograms for glycopolymers and strains respectively.

4.2 Emission change data

4.2.1 Lung pathogen data

Table S26 Dilution corrected relative changes in fluorescence emission intensity for LDA of lung pathogens.

Pathogen	Receptor									
	P3-Glc	P3-Gal	P3-Man	P3-Fuc	P3-Mal	P3-Lac	P3-GlcNAc	P3-GalNAc	P3-Neu5Ac	P3-Neu5Gc
PAO1	1.025	0.966	0.946	0.975	0.970	0.952	1.078	0.997	1.019	0.927
PAO1	1.027	0.970	0.949	0.967	0.955	0.938	1.096	0.998	1.004	0.926
PAO1	1.025	0.971	0.952	0.964	0.947	0.934	1.087	1.025	1.010	0.929
PAO1	1.025	0.966	0.959	0.953	0.946	0.926	1.050	1.002	1.024	0.944
PAO1	1.026	0.978	0.962	0.972	0.942	0.926	1.056	0.975	0.963	0.940
PAO1	1.031	0.952	0.957	0.962	0.941	0.931	1.025	0.994	0.995	0.952
S. aureus	1.013	1.007	0.970	0.978	0.976	0.976	1.025	1.013	1.016	0.954
S. aureus	1.005	1.006	0.969	0.971	0.979	0.976	1.027	1.015	1.002	0.957
S. aureus	1.010	1.005	0.974	0.976	0.981	0.980	1.023	1.014	0.999	0.959
S. aureus	1.008	1.009	0.976	0.978	0.981	0.989	1.030	1.013	1.007	0.964
S. aureus	1.005	1.013	0.975	0.977	0.985	0.989	1.023	1.010	1.005	0.963
S. aureus	1.002	1.004	0.978	0.967	0.978	0.994	1.031	1.001	0.993	0.962
S. maltophilia	1.010	0.995	0.976	0.992	0.981	0.984	1.018	0.990	1.001	0.963
S. maltophilia	1.011	0.994	0.978	0.992	0.984	0.980	1.024	0.992	0.989	0.956
S. maltophilia	1.011	0.997	0.977	0.995	0.983	0.985	1.018	0.988	0.980	0.959
S. maltophilia	1.010	0.996	0.978	0.996	0.989	0.996	1.019	0.989	0.980	0.959
S. maltophilia	1.009	0.998	0.985	0.999	0.995	0.998	1.024	0.993	0.988	0.966
S. maltophilia	1.008	0.995	0.980	0.988	0.992	1.004	1.025	0.983	0.987	0.975
Serratia	1.029	1.013	0.976	1.005	0.988	0.999	1.073	1.018	1.016	0.997
Serratia	1.031	1.011	0.982	1.000	0.980	1.000	1.037	1.023	1.018	0.991
Serratia	1.029	1.013	0.981	0.988	0.982	0.996	1.037	1.017	1.012	0.981
Serratia	1.026	1.017	0.981	0.991	0.980	1.004	1.041	1.018	1.016	0.982
Serratia	1.027	1.011	0.974	0.988	0.976	1.005	1.040	1.012	1.020	0.982
Serratia	1.021	1.018	0.975	0.986	0.975	1.006	1.046	1.013	1.016	0.975

Table S27 Averages and standard deviations (SD) of dilution corrected relative changes in fluorescence emission intensity (I/I0) upon addition of lung pathogens.

Pathogen		Receptor									
		P3-Glc	P3-Gal	P3-Man	P3-Fuc	P3-Mal	P3-Lac	P3-GlcNAc	P3-GalNAc	P3-Neu5Ac	P3-Neu5Gc
**PAO1**	**Mean**	1.027	0.967	0.954	0.965	0.950	0.935	1.065	0.998	1.003	0.937
	**SD**	0.002	0.008	0.006	0.007	0.010	0.009	0.024	0.015	0.020	0.010
S. aureus	**Mean**	1.007	1.007	0.974	0.975	0.980	0.984	1.027	1.011	1.004	0.960
	**SD**	0.004	0.003	0.003	0.004	0.003	0.007	0.003	0.005	0.007	0.003
S. maltophilia	**Mean**	1.010	0.996	0.979	0.993	0.987	0.991	1.021	0.989	0.987	0.963
	**SD**	0.001	0.001	0.003	0.003	0.005	0.009	0.003	0.003	0.007	0.006
Serratia	**Mean**	1.027	1.014	0.978	0.993	0.980	1.002	1.046	1.017	1.016	0.985
	**SD**	0.003	0.003	0.003	0.007	0.004	0.003	0.012	0.004	0.002	0.007

4.2.2 Clinical isolate with 'unknown' lung pathogens data

Table S28 Dilution corrected relative changes in fluorescence emission intensity for LDA of clinical isolates, lung pathogens and a control group.

Isolate	Receptor									
	P3-Glc	P3-Gal	P3-Man	P3-Fuc	P3-Mal	P3-Lac	P3-GlcNAc	P3-GalNAc	P3-Neu5Ac	P3-Neu5gc
PAO1	1.025	0.966	0.946	0.975	0.970	0.952	1.078	0.997	1.019	0.927
PAO1	1.027	0.970	0.949	0.967	0.955	0.938	1.096	0.998	1.004	0.926
PAO1	1.025	0.971	0.952	0.964	0.947	0.934	1.087	1.025	1.010	0.929
PAO1	1.025	0.966	0.959	0.953	0.946	0.926	1.050	1.002	1.024	0.944
PAO1	1.026	0.978	0.962	0.972	0.942	0.926	1.056	0.975	0.963	0.940
PAO1	1.031	0.952	0.957	0.962	0.941	0.931	1.025	0.994	0.995	0.952
DK32	1.159	1.254	1.109	1.243	1.197	1.180	1.221	1.253	1.136	1.027
DK32	1.205	1.258	1.074	1.209	1.172	1.227	1.237	1.265	1.112	1.040
DK32	1.197	1.257	1.073	1.220	1.200	1.213	1.266	1.268	1.153	1.072
DK32	1.216	1.265	0.988	1.238	1.218	1.237	1.287	1.270	1.152	1.067
DK32	1.235	1.299	1.058	1.244	1.202	1.216	1.305	1.314	1.187	1.056
DK32	1.241	1.375	1.024	1.294	1.257	1.318	1.348	1.351	1.187	1.122
DK15	1.406	1.506	1.305	1.491	1.310	1.514	1.671	1.614	1.302	1.341
DK15	1.584	1.513	1.296	1.497	1.293	1.534	1.648	1.616	1.289	1.270
DK15	1.601	1.620	1.318	1.530	1.356	1.559	1.710	1.638	1.311	1.432
DK15	1.619	1.606	1.337	1.563	1.376	1.590	1.677	1.645	1.285	1.382
DK15	1.631	1.649	1.290	1.577	1.419	1.611	1.744	1.755	1.313	1.480
DK15	1.562	1.682	1.423	1.650	1.443	1.628	1.874	1.789	1.322	1.558
DK09	1.112	1.138	1.134	1.139	1.124	1.184	1.163	1.133	1.128	1.146
DK09	1.120	1.154	1.098	1.129	1.019	1.128	1.129	1.125	1.131	1.193
DK09	1.145	1.143	1.090	1.139	1.141	1.150	1.184	1.155	1.133	1.161
DK09	1.145	1.163	1.106	1.139	1.118	1.127	1.215	1.163	1.132	1.148
DK09	1.150	1.172	1.089	1.164	1.058	1.153	1.215	1.146	1.152	1.153
DK09	1.154	1.154	1.096	1.158	1.036	1.149	1.226	1.145	1.142	1.271

DK28	1.221	1.308	0.916	1.304	1.237	1.364	1.406	1.450	1.143	0.974
DK28	1.265	1.313	0.814	1.259	1.212	1.362	1.423	1.419	1.157	1.020
DK28	1.249	1.305	0.840	1.292	1.244	1.327	1.442	1.427	1.144	1.025
DK28	1.197	1.224	0.814	1.280	1.234	1.362	1.460	1.439	1.167	0.895
DK28	1.208	1.330	0.829	1.286	1.271	1.330	1.512	1.443	1.176	1.014
DK28	1.327	1.714	0.875	1.415	1.315	1.449	1.601	1.514	1.186	0.897
DK29	1.103	1.090	0.975	1.106	1.040	1.108	1.140	1.619	1.105	0.875
DK29	1.082	1.080	0.877	1.083	1.082	1.148	1.135	1.093	1.127	0.963
DK29	1.145	1.094	0.888	1.100	1.106	1.107	1.162	1.762	1.135	0.976
DK29	1.097	1.071	0.796	1.100	1.123	1.096	1.261	1.086	1.134	0.964
DK29	1.197	1.086	0.875	1.099	1.095	1.119	1.170	1.132	1.162	0.942
DK29	1.814	1.102	1.008	1.162	1.105	1.120	1.176	1.259	1.157	0.960
control	1.034	0.990	0.947	1.000	1.014	0.995	0.968	1.001	0.989	0.969
control	1.003	1.020	1.012	0.986	0.990	0.992	0.966	0.996	0.979	0.972
control	0.995	0.986	1.016	0.998	1.005	1.001	1.021	0.992	0.988	1.037
control	0.991	0.984	0.999	0.994	0.996	1.014	0.983	0.988	1.016	1.012
control	0.988	1.010	0.995	1.023	1.003	0.994	1.026	1.014	1.009	1.022
control	0.988	1.009	1.031	0.999	0.992	1.004	1.035	1.009	1.020	0.989
S. aureus	1.013	1.007	0.970	0.978	0.976	0.976	1.025	1.013	1.016	0.954
S. aureus	1.005	1.006	0.969	0.971	0.979	0.976	1.027	1.015	1.002	0.957
S. aureus	1.010	1.005	0.974	0.976	0.981	0.980	1.023	1.014	0.999	0.959
S. aureus	1.008	1.009	0.976	0.978	0.981	0.989	1.030	1.013	1.007	0.964
S. aureus	1.005	1.013	0.975	0.977	0.985	0.989	1.023	1.010	1.005	0.963
S. aureus	1.002	1.004	0.978	0.967	0.978	0.994	1.031	1.001	0.993	0.962
S. maltophilia	1.010	0.995	0.976	0.992	0.981	0.984	1.018	0.990	1.001	0.963
S. maltophilia	1.011	0.994	0.978	0.992	0.984	0.980	1.024	0.992	0.989	0.956
S. maltophilia	1.011	0.997	0.977	0.995	0.983	0.985	1.018	0.988	0.980	0.959
S. maltophilia	1.010	0.996	0.978	0.996	0.989	0.996	1.019	0.989	0.980	0.959
S. maltophilia	1.009	0.998	0.985	0.999	0.995	0.998	1.024	0.993	0.988	0.966
S. maltophilia	1.008	0.995	0.980	0.988	0.992	1.004	1.025	0.983	0.987	0.975
Serratia	1.029	1.013	0.976	1.005	0.988	0.999	1.073	1.018	1.016	0.997
Serratia	1.031	1.011	0.982	1.000	0.980	1.000	1.037	1.023	1.018	0.991
Serratia	1.029	1.013	0.981	0.988	0.982	0.996	1.037	1.017	1.012	0.981
Serratia	1.026	1.017	0.981	0.991	0.980	1.004	1.041	1.018	1.016	0.982
Serratia	1.027	1.011	0.974	0.988	0.976	1.005	1.040	1.012	1.020	0.982
Serratia	1.021	1.018	0.975	0.986	0.975	1.006	1.046	1.013	1.016	0.975

4.3 LDA canonical discriminant function coefficients

Table S29 Canonical discriminant function coefficients for receptors obtained through LDA.

	Function		
	**F1**	**F2**	**F3**
**P3-Glc**	232.504	459.841	18.537
**P3-Gal**	309.986	78.766	-141.787
**P3-Man**	-159.826	-78.480	254.368
**P3-Fuc**	-67.090	-145.121	183.040
**P3-Mal**	13.816	-72.456	-118.745
**P3-Lac**	110.760	67.293	71.487
**P3-GlcNAc**	-55.077	57.030	29.643
**P3-GalNAc**	61.515	-30.562	-71.423
**P3-Neu5Ac**	-12.693	-2.928	63.741
**P3-Neu5Gc**	113.793	40.081	-75.994
**(Constant)**	-547.132	-387.127	-208.618

4.4 PERMANOVA analysis

Table S30 Full results of pairwise PERMANOVA (adonis2) analysis of the lung pathogens dataset.

**Pairs**	**Df**	**Sums Of Squares**	**F Model**	R^2	***p***	***p* adjusted**
PAO1 vs *S. aureus*	1	0.02402	21.92	0.69	0.004	0.016
PAO1 vs *S. maltophilia*	1	0.030184	27.08	0.73	0.001	0.006
PAO1 vs *Serratia*	1	0.036517	31	0.76	0.008	0.016
S. aureus vs *S. maltophilia*	1	0.004245	16.31	0.62	0.004	0.016
S. aureus vs serratia	1	0.006886	21.27	0.68	0.005	0.016
S. maltophilia vs *Serratia*	1	0.010337	30.19	0.75	0.001	0.006

4.5 Confusion matrix

Table S31 Confusion matrix detailing results of cross-validation of lung pathogen LDA model using 10-glycopolymer array.

		**Predicted group**			
		PAO1	*S. aureus*	*S. maltophilia*	*Serratia*
**Actual group**	PAO1	6	0	0	0
	S. aureus	0	5	1	0
	S. maltophilia	0	1	5	0
	Serratia	0	0	0	6

4.6 Hold-out validation

Hold-out validation was conducted on the dataset using 4 datapoints for each pathogen (Table S32, green) to construct an LDA scoring model which was used to classify the remaining datapoints for each

strain (Table S32, red). The hold-out dataset was classified with 88% accuracy (Table 33; 7/8), corresponding to a single misclassification of *S. aureus* as *S. maltophilia*.

Table S32 Dilution corrected relative changes in fluorescence emission intensity (I/I_0) upon addition of lung pathogens, with data used for training model highlighted in green and the hold-out set in red.

Mutant	Receptor									
	P3-Glc	P3-Gal	P3-Man	P3-Fuc	P3-Mal	P3-Lac	P3-GlcNAc	P3-GalNAc	P3-Neu5Ac	P3-Neu5Gc
PAO1	1.025	0.966	0.946	0.975	0.970	0.952	1.078	0.997	1.019	0.927
PAO1	1.027	0.970	0.949	0.967	0.955	0.938	1.096	0.998	1.004	0.926
PAO1	1.025	0.971	0.952	0.964	0.947	0.934	1.087	1.025	1.010	0.929
PAO1	1.025	0.966	0.959	0.953	0.946	0.926	1.050	1.002	1.024	0.944
PAO1	1.026	0.978	0.962	0.972	0.942	0.926	1.056	0.975	0.963	0.940
PAO1	1.031	0.952	0.957	0.962	0.941	0.931	1.025	0.994	0.995	0.952
S. aureus	1.013	1.007	0.970	0.978	0.976	0.976	1.025	1.013	1.016	0.954
S. aureus	1.005	1.006	0.969	0.971	0.979	0.976	1.027	1.015	1.002	0.957
S. aureus	1.010	1.005	0.974	0.976	0.981	0.980	1.023	1.014	0.999	0.959
S. aureus	1.008	1.009	0.976	0.978	0.981	0.989	1.030	1.013	1.007	0.964
S. aureus	1.005	1.013	0.975	0.977	0.985	0.989	1.023	1.010	1.005	0.963
S. aureus	1.002	1.004	0.978	0.967	0.978	0.994	1.031	1.001	0.993	0.962
S. maltophilia	1.010	0.995	0.976	0.992	0.981	0.984	1.018	0.990	1.001	0.963
S. maltophilia	1.011	0.994	0.978	0.992	0.984	0.980	1.024	0.992	0.989	0.956
S. maltophilia	1.011	0.997	0.977	0.995	0.983	0.985	1.018	0.988	0.980	0.959
S. maltophilia	1.010	0.996	0.978	0.996	0.989	0.996	1.019	0.989	0.980	0.959
S. maltophilia	1.009	0.998	0.985	0.999	0.995	0.998	1.024	0.993	0.988	0.966
S. maltophilia	1.008	0.995	0.980	0.988	0.992	1.004	1.025	0.983	0.987	0.975
Serratia	1.029	1.013	0.976	1.005	0.988	0.999	1.073	1.018	1.016	0.997
Serratia	1.031	1.011	0.982	1.000	0.980	1.000	1.037	1.023	1.018	0.991
Serratia	1.029	1.013	0.981	0.988	0.982	0.996	1.037	1.017	1.012	0.981
Serratia	1.026	1.017	0.981	0.991	0.980	1.004	1.041	1.018	1.016	0.982
Serratia	1.027	1.011	0.974	0.988	0.976	1.005	1.040	1.012	1.020	0.982
Serratia	1.021	1.018	0.975	0.986	0.975	1.006	1.046	1.013	1.016	0.975

Table S33 Confusion matrix detailing results of hold-out validation of lung pathogen dataset.

		Predicted group			
		PAO1	*S. aureus*	*S. maltophilia*	*Serratia*
**Actual group**	PAO1	2	0	0	0
	S. aureus	0	1	1	0
	S. maltophilia	0	0	2	0
	Serratia	0	0	0	2

4.7 3D plot of LDA from Figure 3c

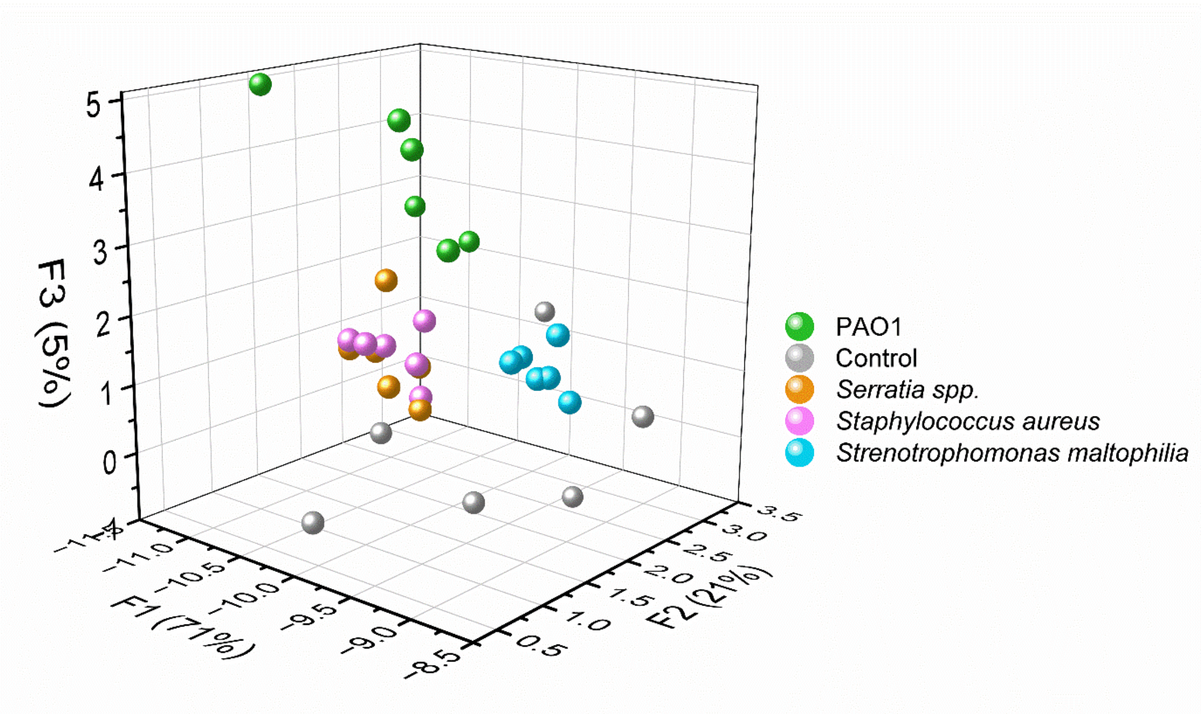

Figure S9 Partial 3D canonical LDA score plot for functions 1, 2 and 3, highlighting discrimination between PAO1 and the control group in 3D space.

4.8 Principal component analysis (PCA) results

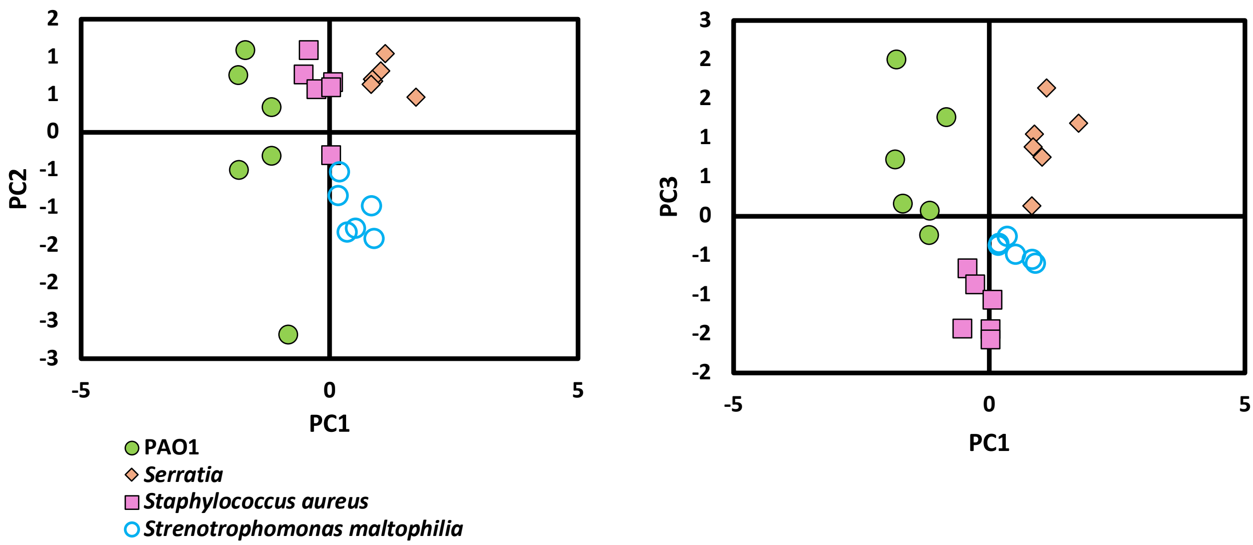

Figure S10 PCA score plots for functions 1 & 2 and functions 1 & 3 for the analysis of the lung pathogens performed in sextuplicate (5.0 µM receptors, pH 7.4).

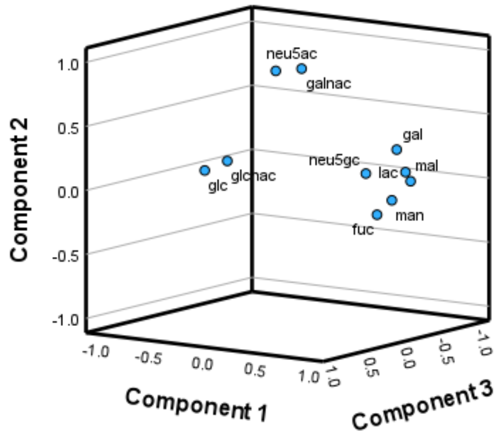

Figure S11 PCA loading plot visualising relationship between glycopolymers and principal components for the lung pathogen dataset.

4.9 LDA including PAO1 dilutions

To assess the response of the sensor array to analytes at different effective concentrations, PAO1 was grown to saturation from a glycerol stock (stored at -80 °C) in Luria–Bertani medium (10 g L^{-1} tryptone, 10 g L^{-1} NaCl, 5 g L^{-1} yeast) at 37 °C for 24 h, maintaining agitation at 180 rpm. The culture was centrifuged (2500 *g*, 5 min.) to pellet the cells, which were resuspended in sterile PBS pH 7.4 (5 mL). Aliquots of this suspension (1 mL) were then diluted with PBS pH 7.4 (1 mL or 3 mL) to achieve 1 in 2 and 1 in 4 dilutions respectively. PAO1 dilutions were included as ungrouped cases and assigned by the LDA model in SPSS. Dilutions were scored as PAO1 in 0/12 cases (Table S35, 0%).

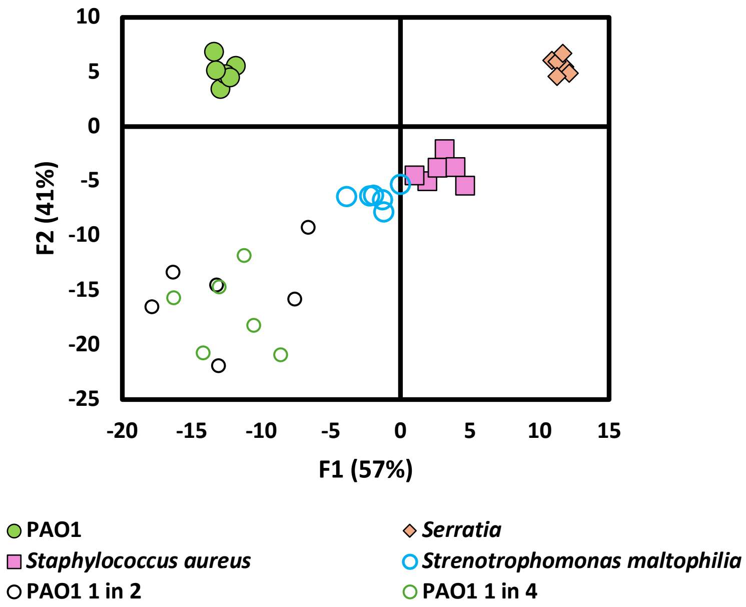

Figure S12 Canonical LDA score plots for functions 1 & 2 for the analysis of the lung pathogens and dilutions of PAO1 performed in sextuplicate (5.0 µM receptors, pH 7.4).

Table S34 Dilution corrected data for PAO1 dilutions in PBS.

Dilution	Receptor									
	P3-Glc	P3-Gal	P3-Man	P3-Fuc	P3-Mal	P3-Lac	P3-GlcNAc	P3-GalNAc	P3-Neu5Ac	P3-Neu5Gc
**1 in 2**	0.994	0.969	0.978	0.973	0.991	0.981	1.041	0.991	1.021	0.948
**1 in 2**	0.990	0.978	0.988	0.987	0.991	0.976	1.053	0.987	1.026	0.953
**1 in 2**	0.992	0.984	0.991	0.979	0.990	0.981	1.039	0.986	1.020	0.963
**1 in 2**	0.998	0.991	1.000	0.956	0.990	0.986	1.019	0.994	1.003	0.967
**1 in 2**	0.998	0.992	1.000	0.989	0.989	0.977	0.989	0.991	1.012	0.972
**1 in 2**	0.984	0.989	1.000	0.991	0.993	0.974	1.006	0.983	1.019	0.976
**1 in 4**	1.003	0.979	0.985	0.983	0.991	0.978	1.012	0.986	0.996	0.951
**1 in 4**	0.989	0.982	0.988	0.986	0.992	0.979	1.061	0.981	0.996	0.959
**1 in 4**	0.991	0.987	0.987	0.982	0.990	0.972	1.043	0.981	0.988	0.964
**1 in 4**	0.986	0.981	0.996	0.989	0.986	0.973	1.001	0.984	0.984	0.973
**1 in 4**	0.988	0.992	0.993	0.987	0.989	0.972	0.967	0.977	0.982	0.973
**1 in 4**	0.995	0.982	0.995	0.989	0.988	0.969	0.975	0.976	0.988	0.979

Table S35 Confusion matrix detailing results of LDA after including dilutions of PAO1 as ungrouped cases.

		Predicted group			
		PAO1	*S. aureus*	*S. maltophilia*	*Serratia*
**Actual group**	PAO1	6	0	0	0
	S. aureus	0	6	0	0
	S. maltophilia	0	0	6	0
	Serratia	0	0	0	6
	1 in 2	0	0	6	0
	1 in 4	0	0	6	0

5. NMR Spectra

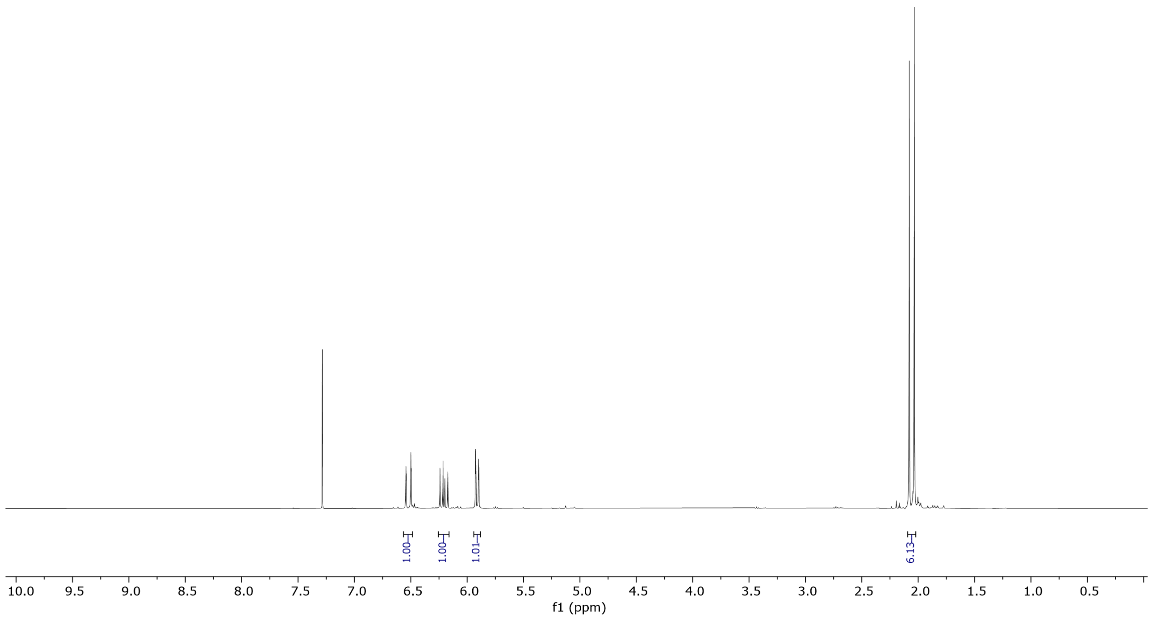

Figure S13 ^{1}H NMR spectrum (400 MHz, $CDCl_3$) of acetone oxime acrylate (**M1**).

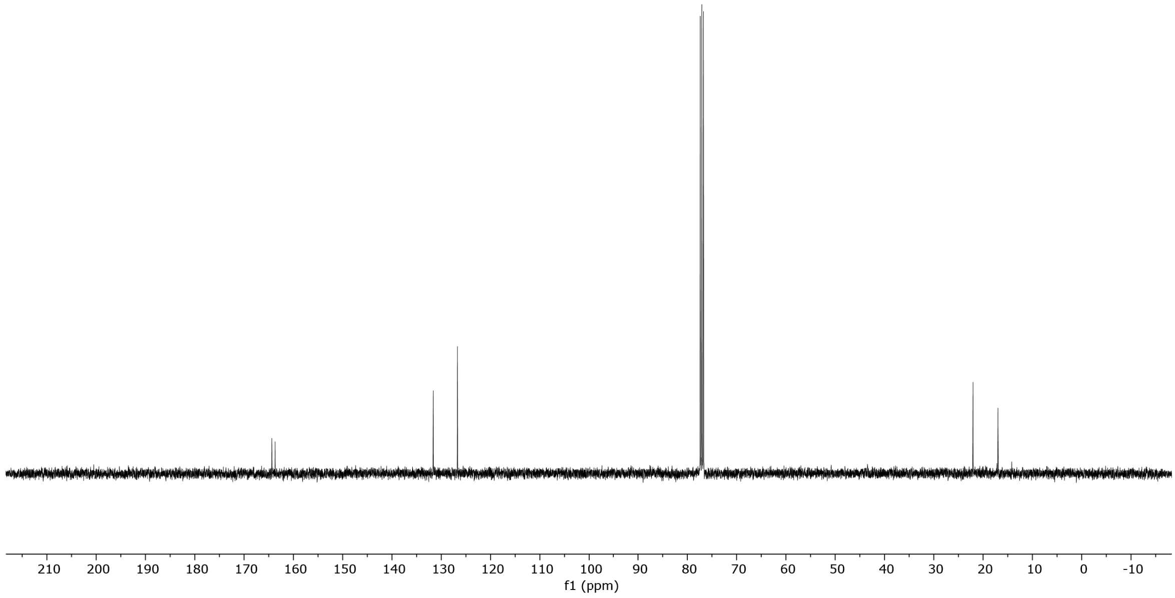

Figure S14 ^{13}C NMR spectrum (101 MHz, $CDCl_3$) of acetone oxime acrylate (**M1**).

2

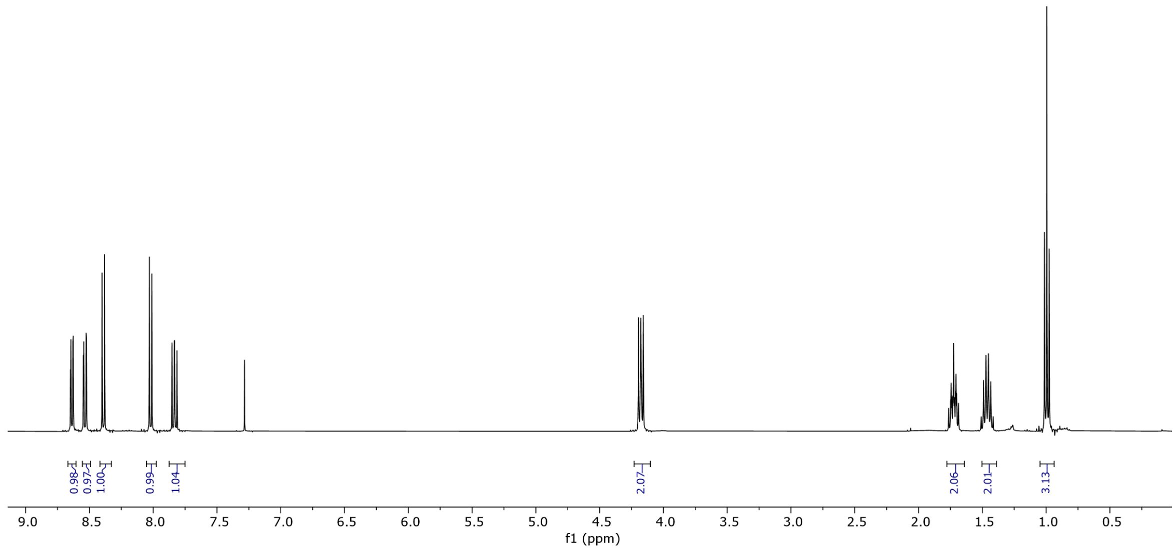

Figure S15 ^{1}H NMR spectrum (400 MHz, $CDCl_3$) of *N*-butyl-4-bromo-1,8-naphthalimide (**2**).

2

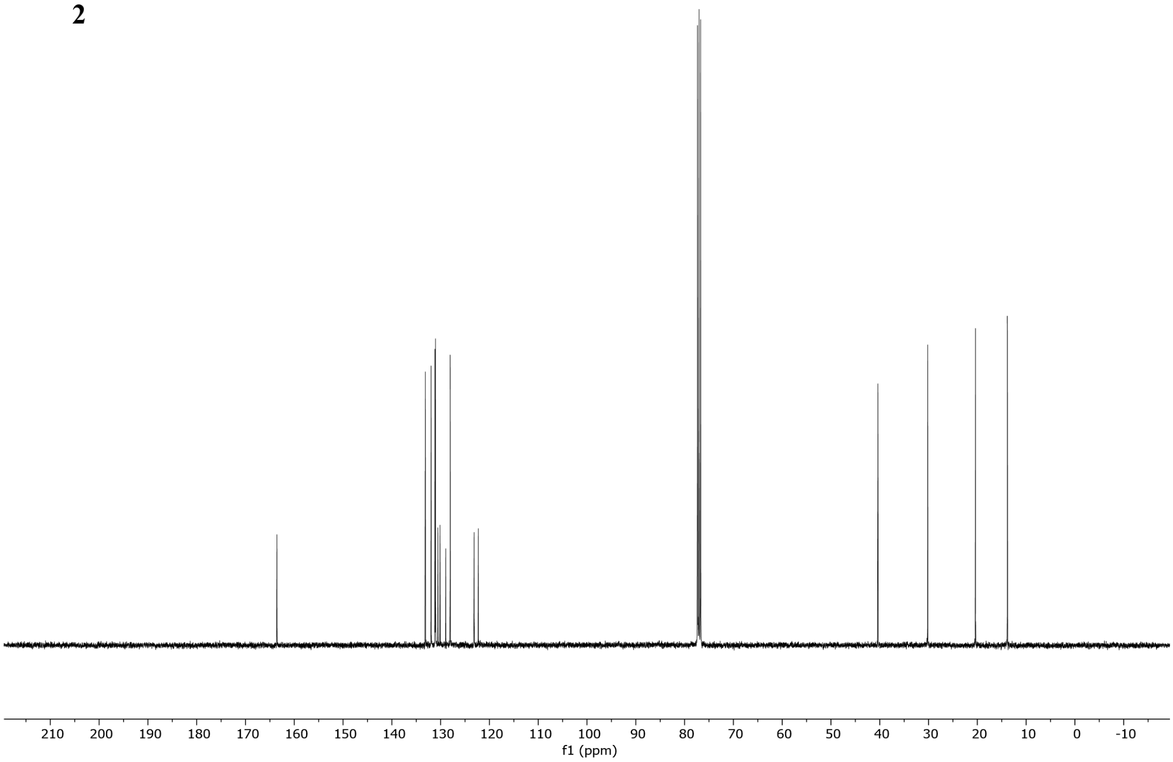

Figure S16 ^{13}C NMR spectrum (101 MHz, $CDCl_3$) of *N*-butyl-4-bromo-1,8-naphthalimide (**2**).

3

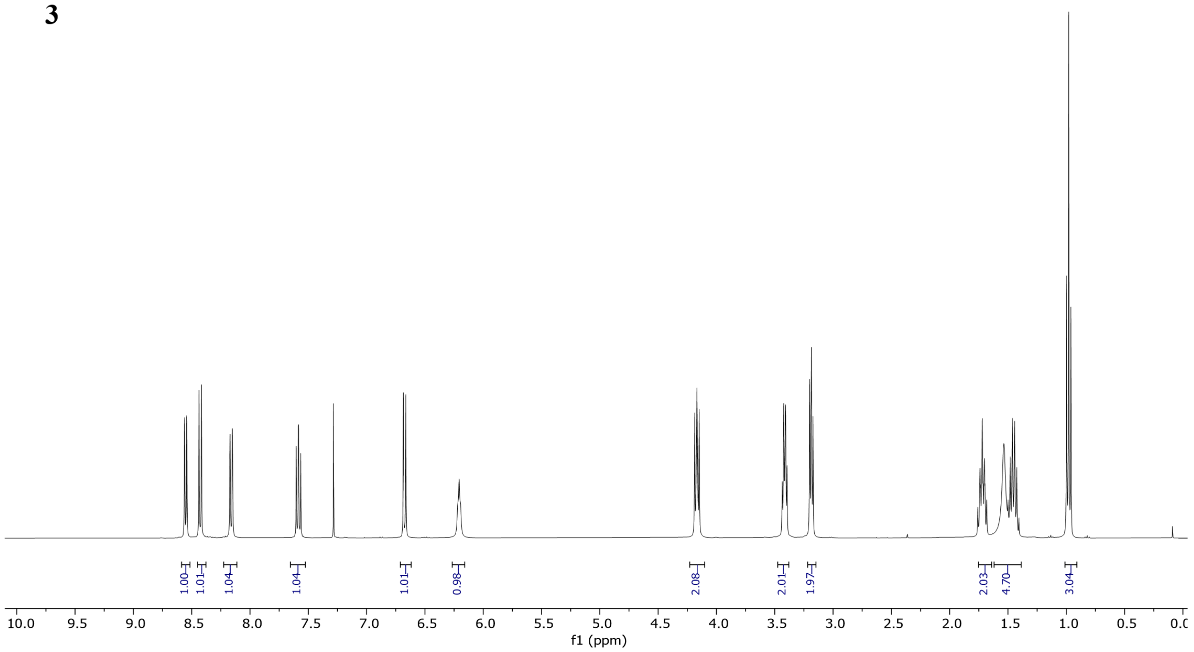

Figure S17 ^{1}H NMR spectrum (400 MHz, CDCl3) of *N*-butyl-4-ethylenediamine-1,8-naphthalimide (**3**)

3

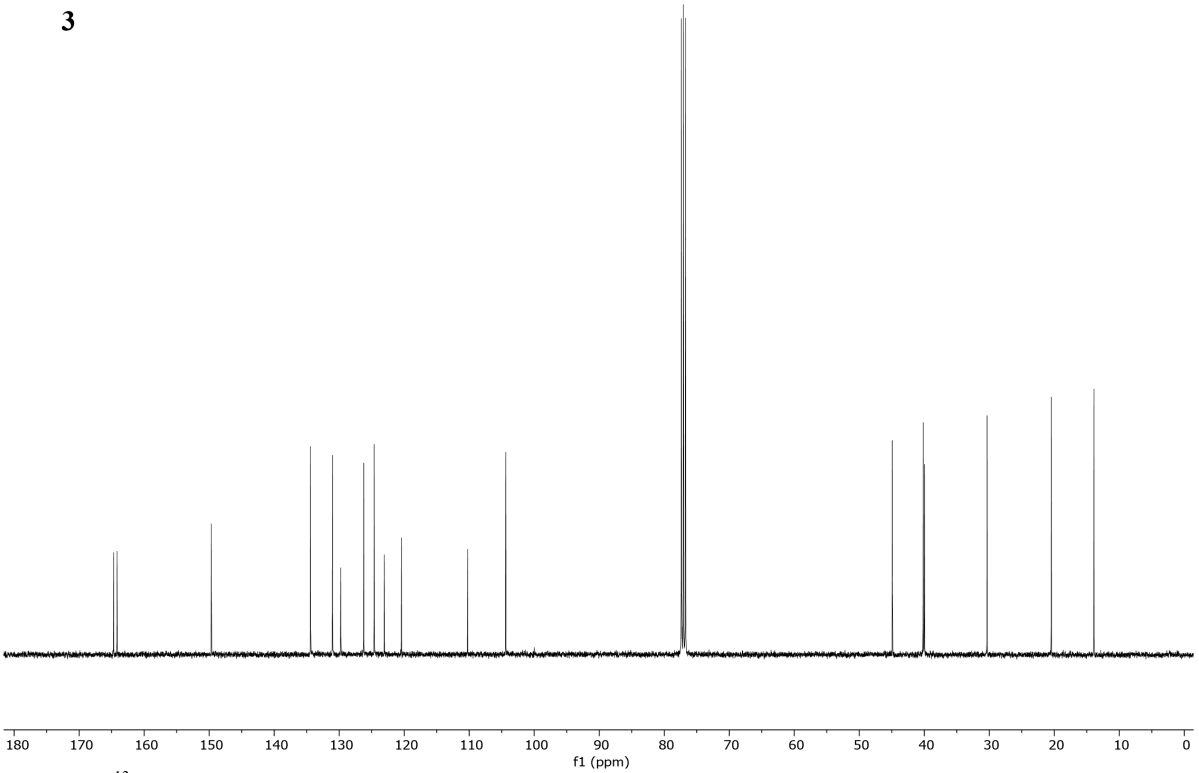

Figure S18 ^{13}C NMR spectrum (101 MHz, $CDCl_3$) of *N*-butyl-4-ethylenediamine-1,8-naphthalimide (**3**).

1

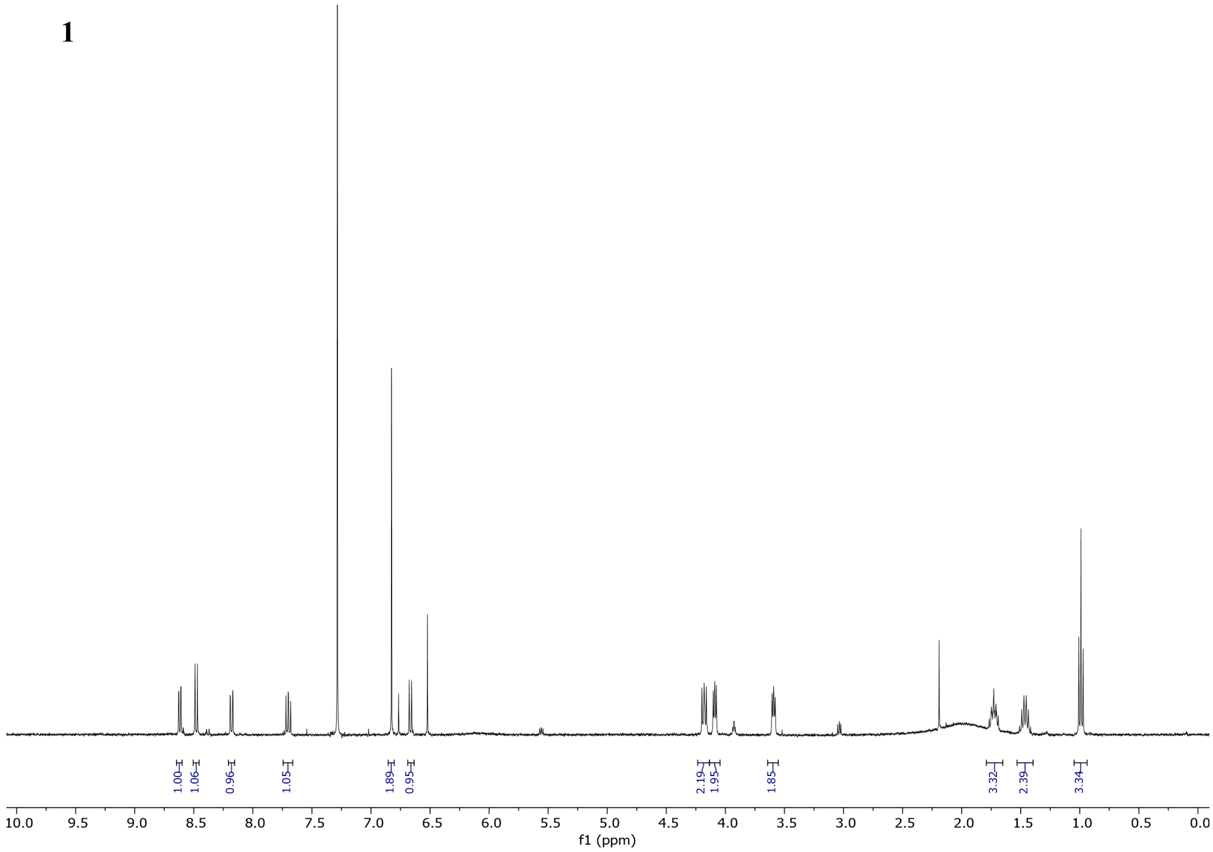

Figure S19 ^{1}H NMR spectrum (400 MHz, $CDCl_3$) of *N*-butyl-4-(2-aminoethyl)-maleimide-1,8-naphthalimide (**1**).

1

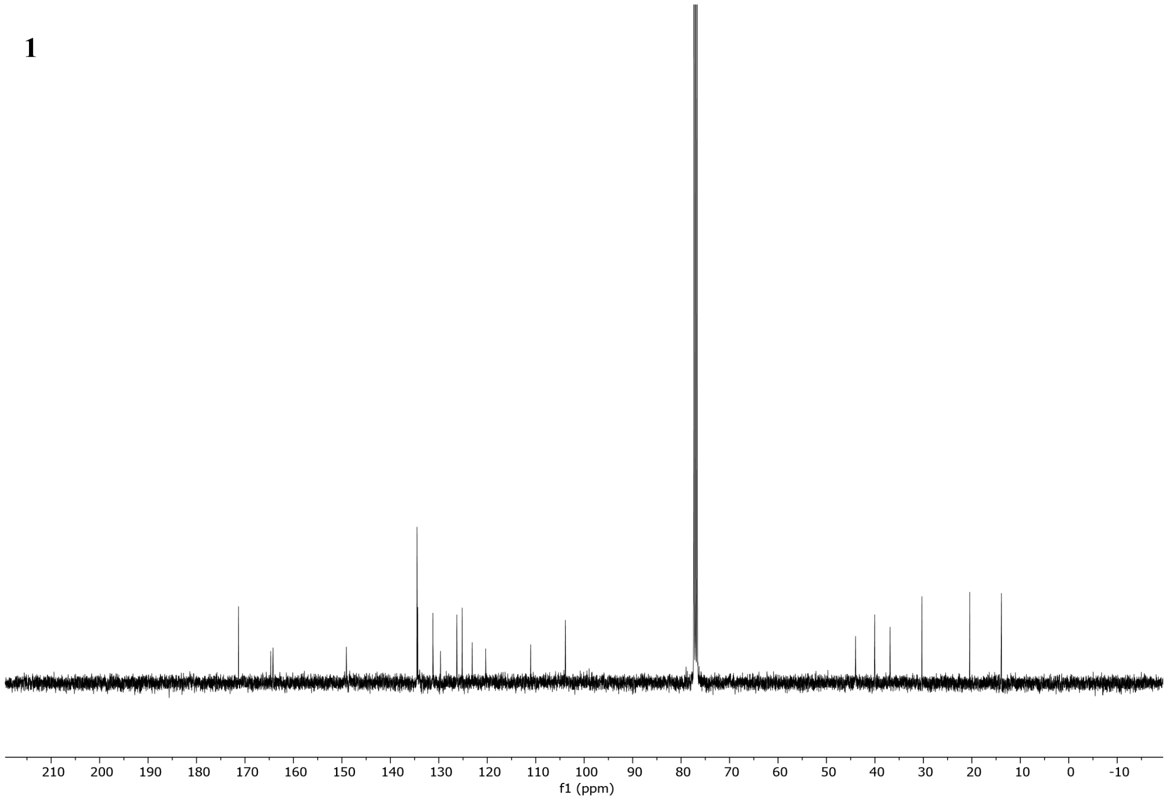

Figure S20 ^{13}C NMR spectrum (101 MHz, $CDCl_3$) of *N*-butyl-4-(2-aminoethyl)-maleimide-1,8-naphthalimide (**1**).

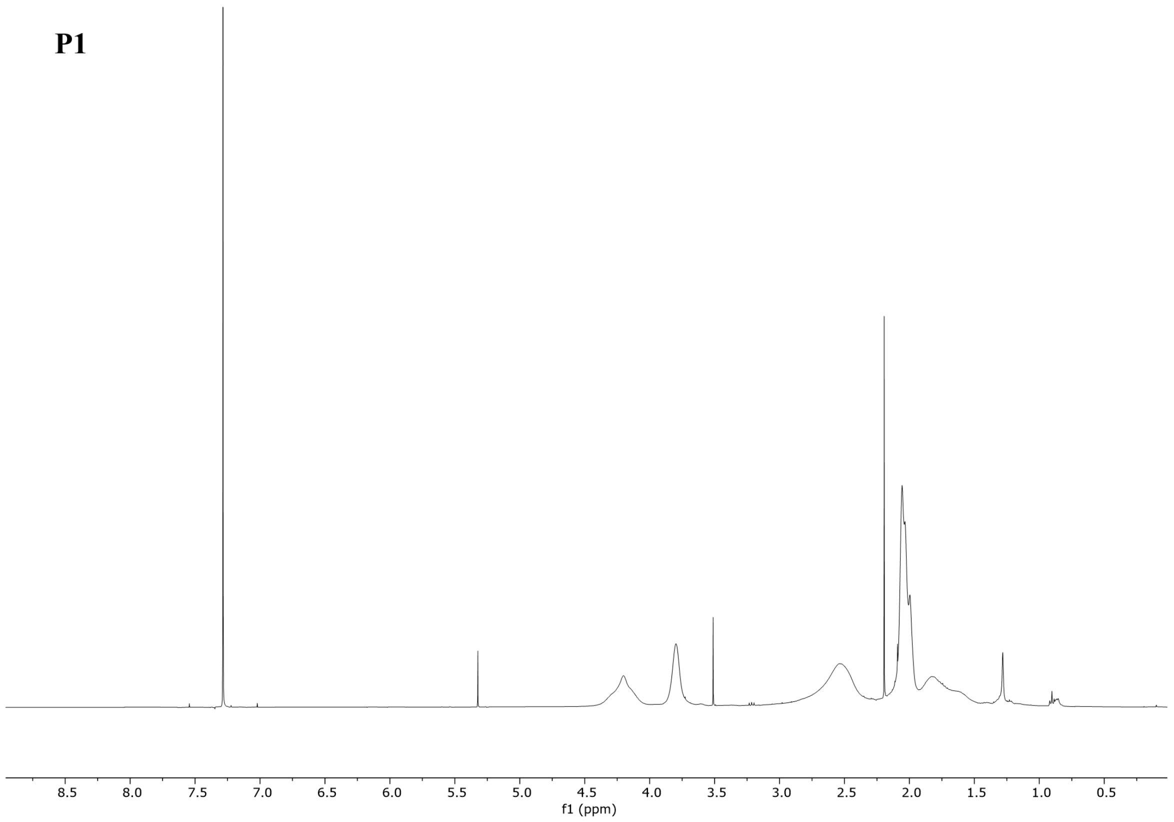

Figure S21 ^{1}H NMR spectrum (400 MHz, $CDCl_3$) of poly(acetone oxime acrylate-stat-hydroxyethyl acrylate) (**P1**).

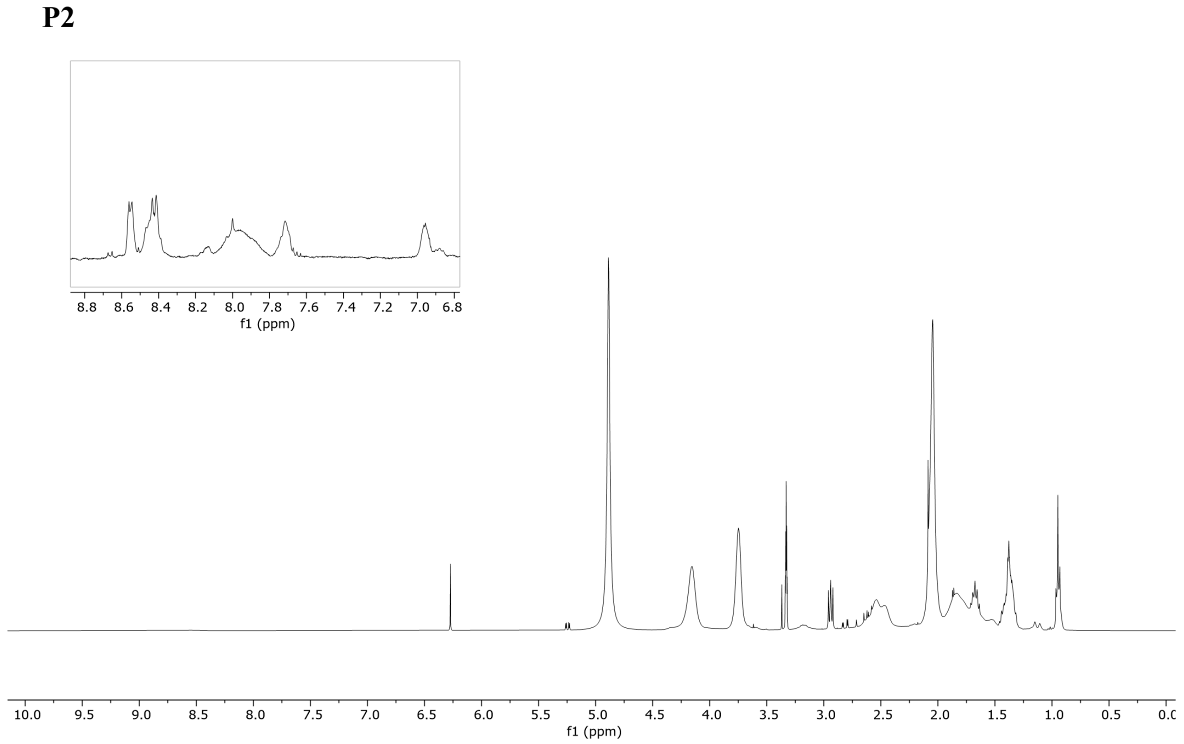

Figure S22 ^{1}H NMR spectrum (400 MHz, MeOH-d_4) of napthalimide-labelled-poly(acetone oxime acrylate-*stat*-hydroxyethyl acrylate) (**P2**).

P3

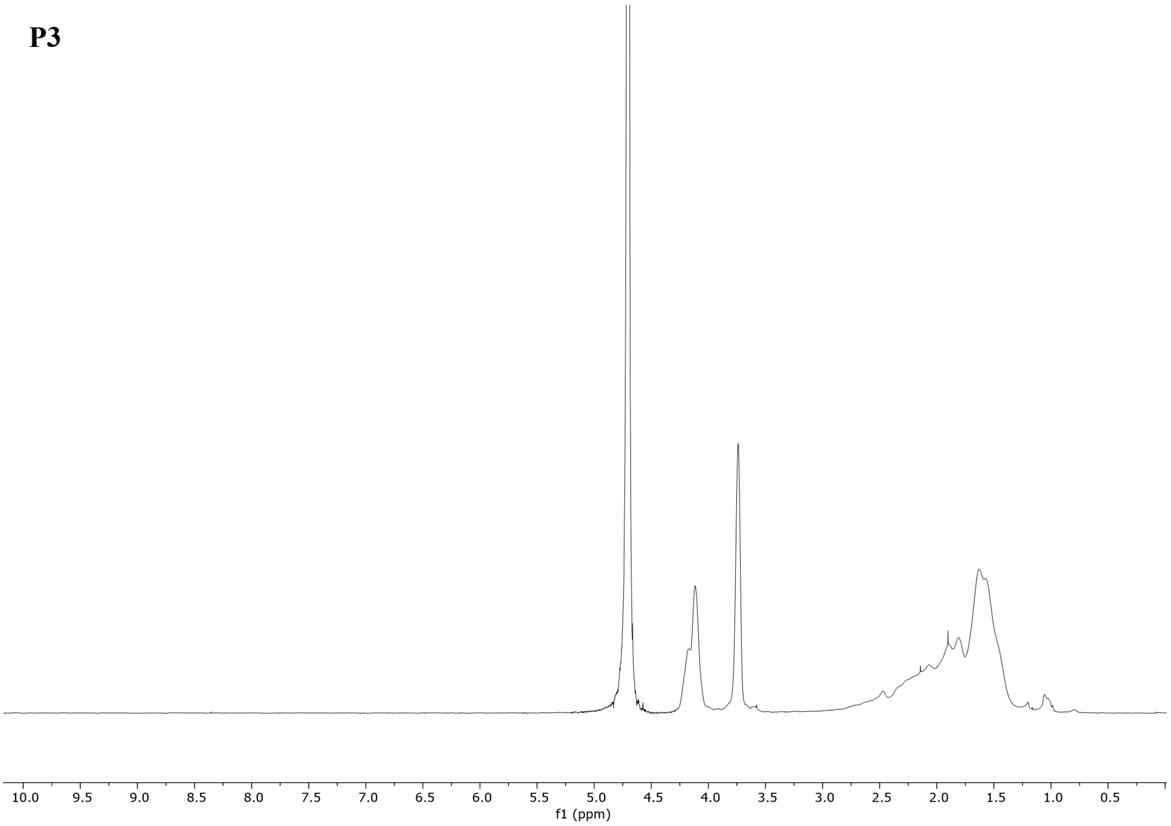

Figure S23 ^{1}H NMR spectrum (400 MHz, D_2O) of napthalimide-labelled-poly(acyl hydrazide-stat-hydroxy ethyl acrylate) (**P3**).

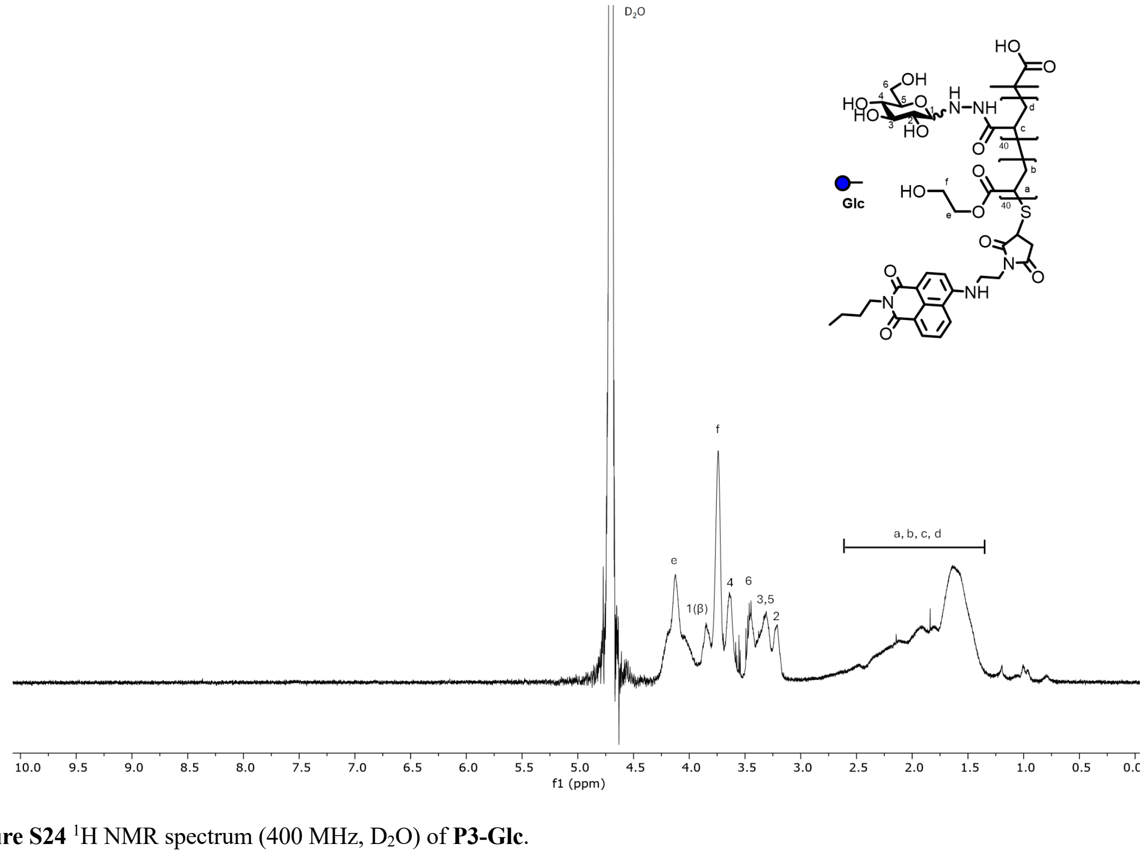

Figure S24 ^{1}H NMR spectrum (400 MHz, D_2O) of **P3-Glc**.

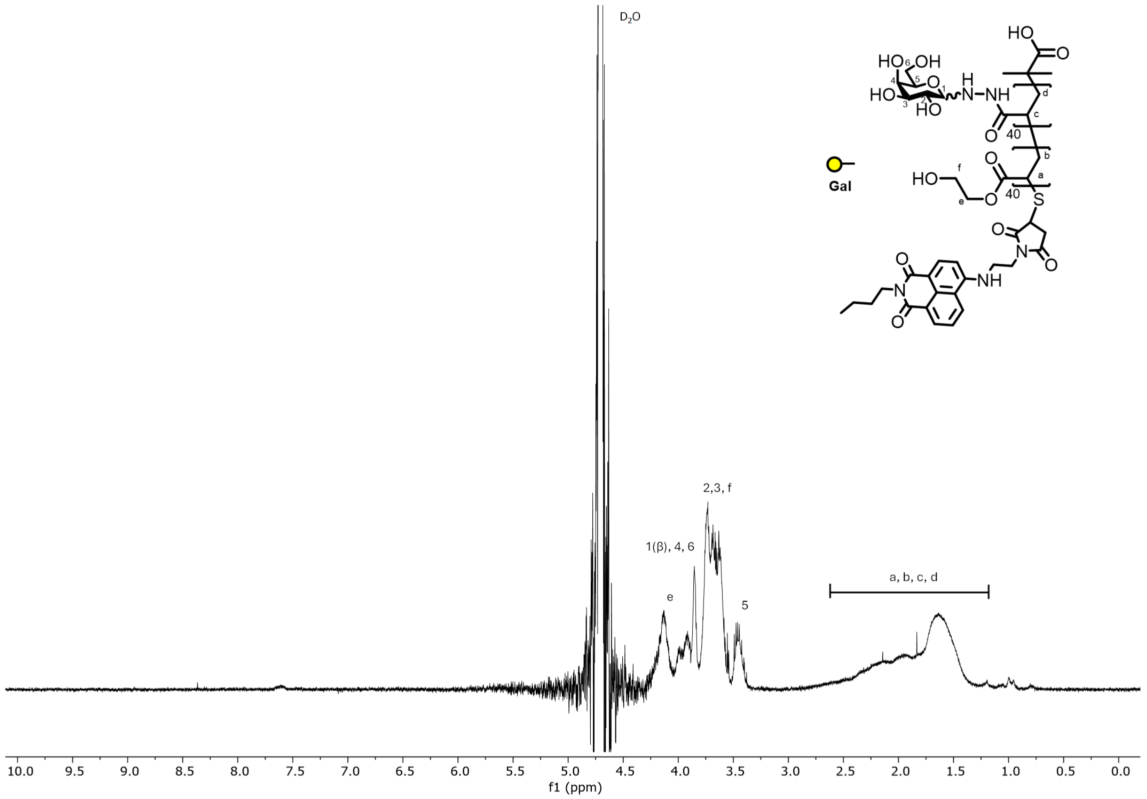

Figure S25 ^{1}H NMR spectrum (400 MHz, D_2O) of **P3-Gal**.

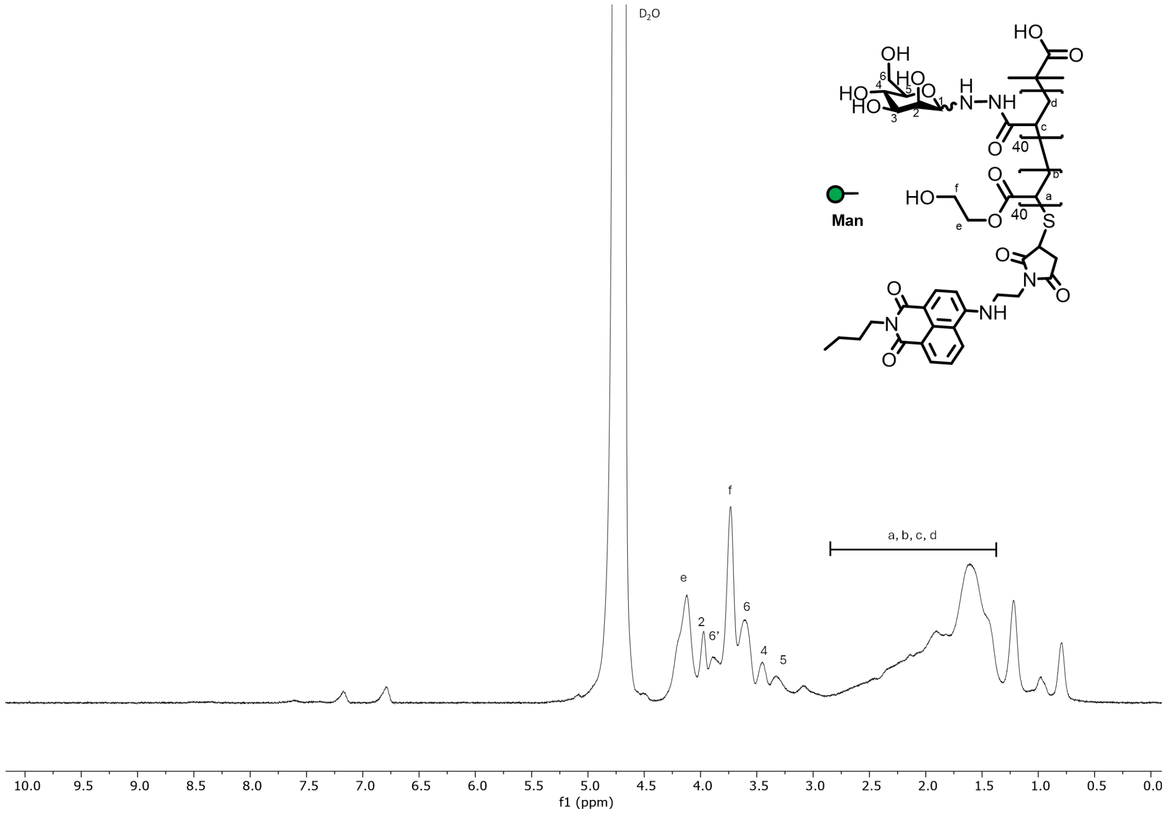

Figure S26 ^{1}H NMR spectrum (400 MHz, D_2O) of **P3-Man**.

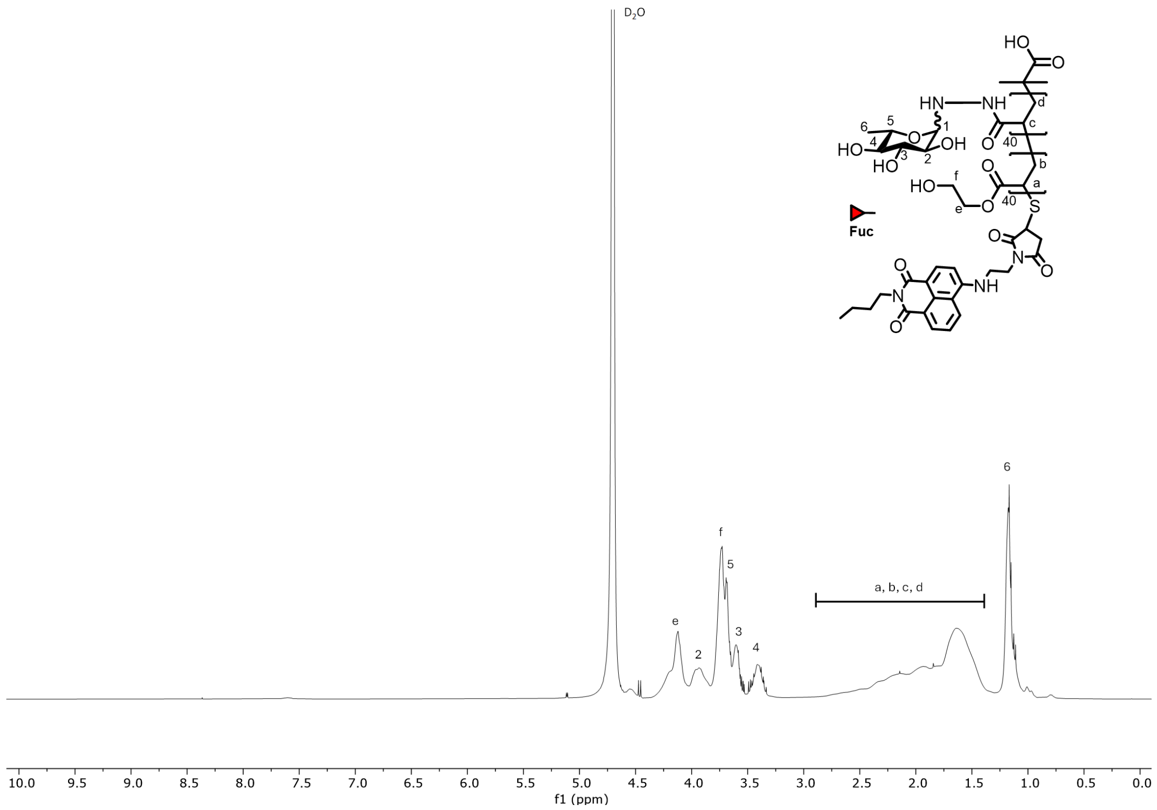

Figure S27 ^{1}H NMR spectrum (400 MHz, D$_2$O) of **P3-Gal**.

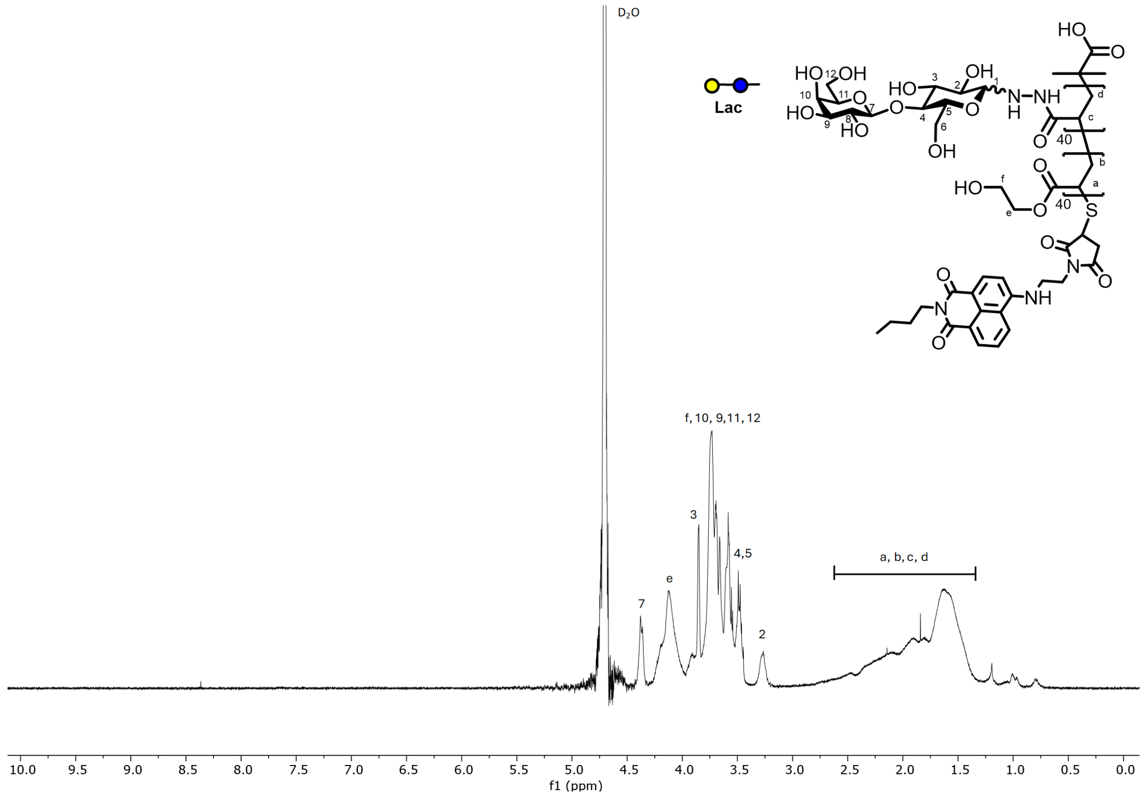

Figure S28 ^{1}H NMR spectrum (400 MHz, D$_2$O) of **P3-Lac**.

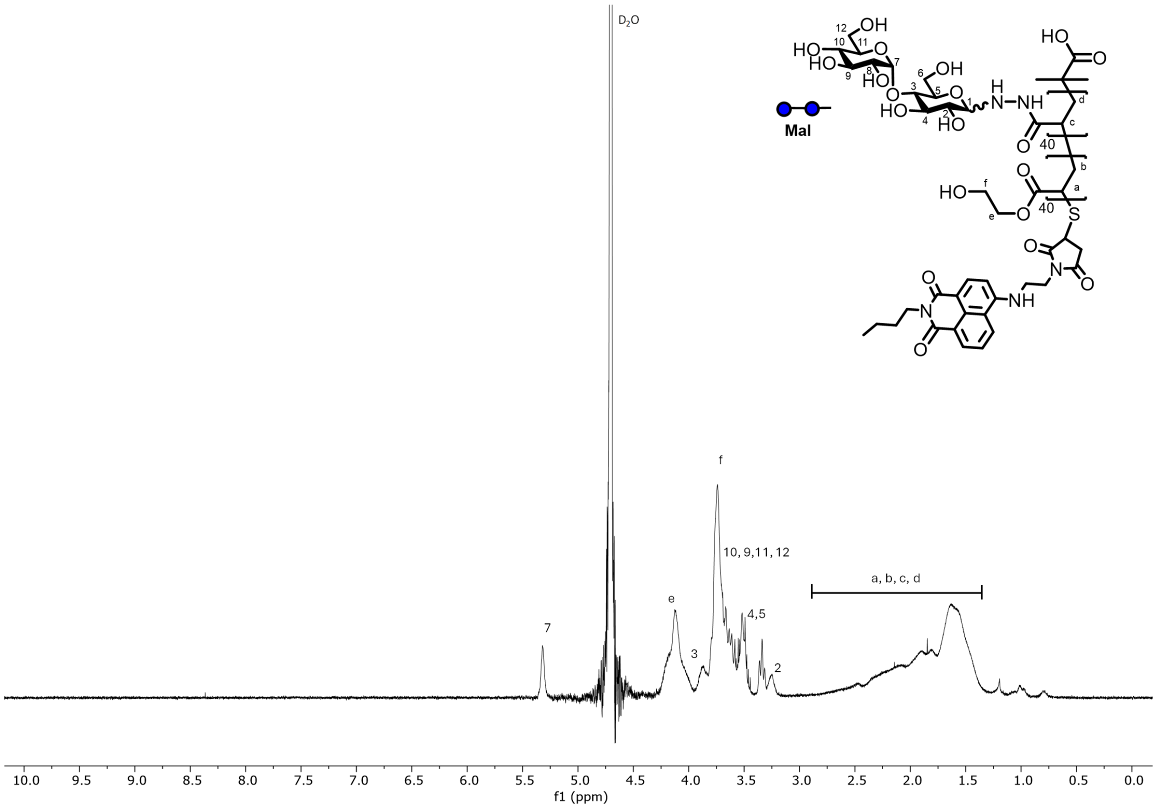

Figure S29 ^{1}H NMR spectrum (400 MHz, D_2O) of **P3-Mal**.

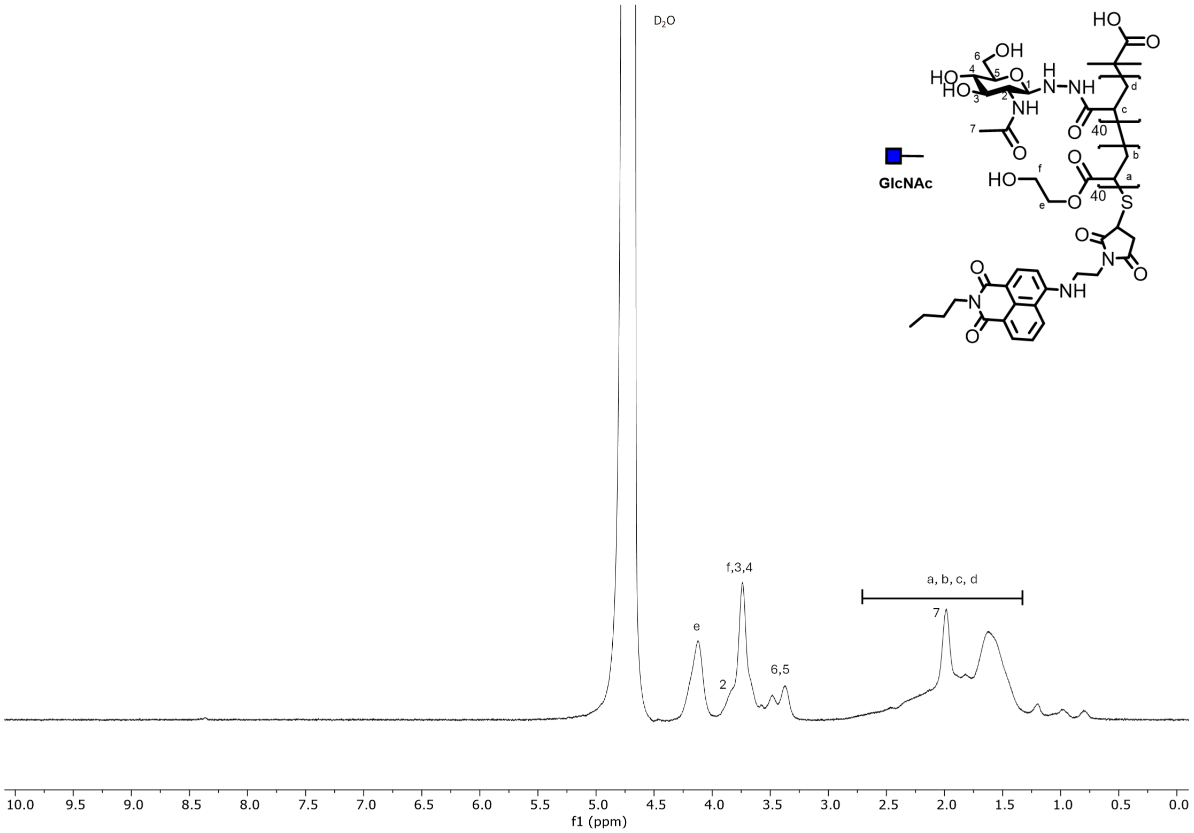

Figure S30 ^{1}H NMR spectrum (400 MHz, D_2O) of **P3-GlcNAc**.

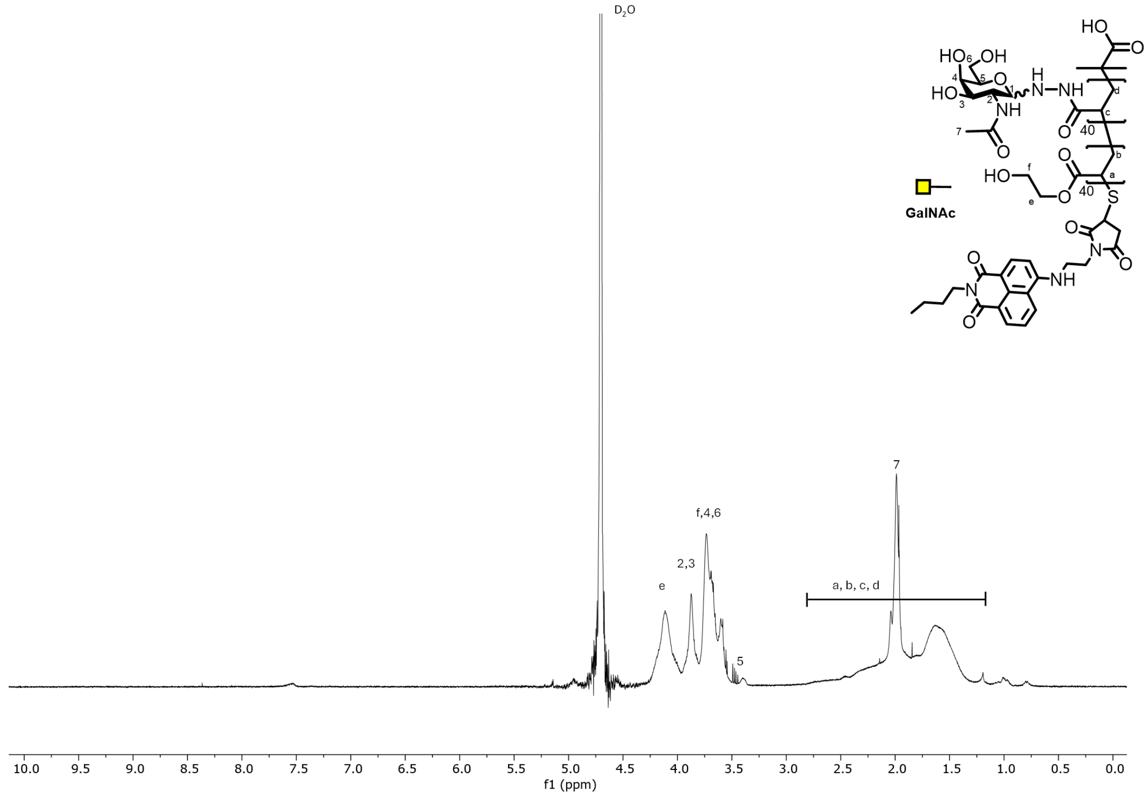

Figure S31 ^{1}H NMR spectrum (400 MHz, D_2O) of **P3-GalNAc**.

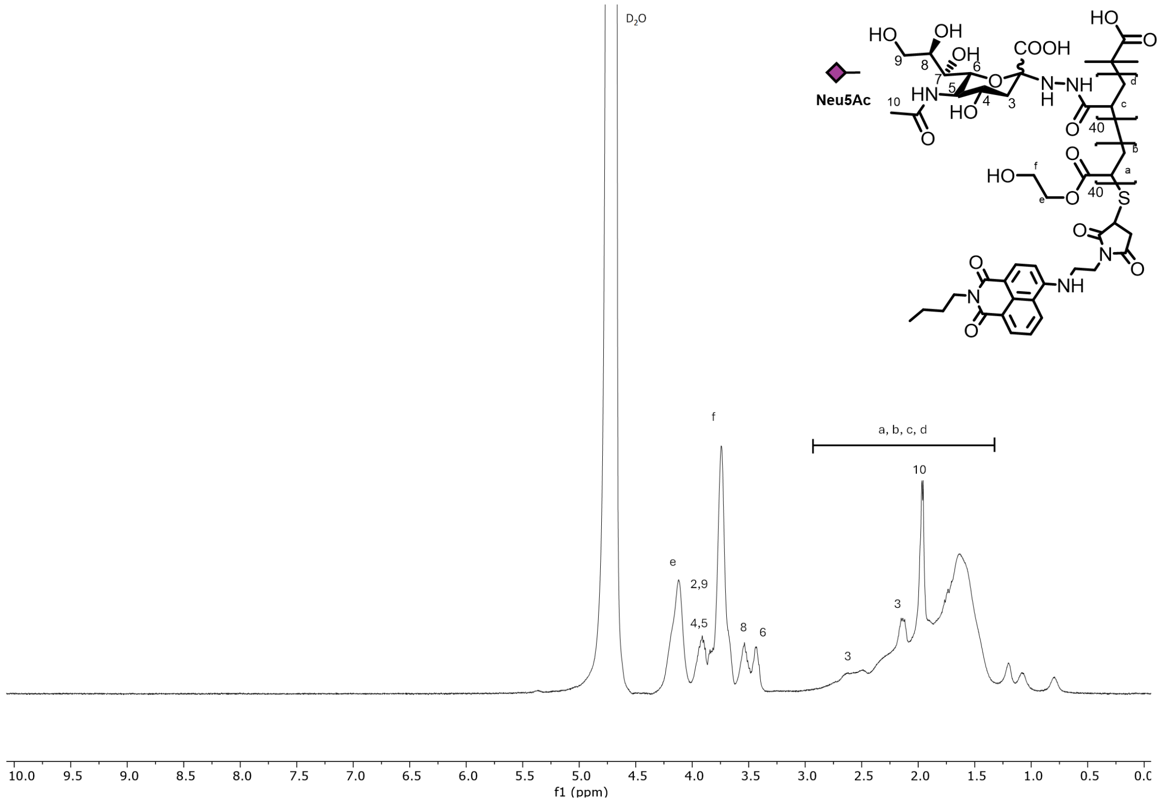

Figure S32 ^{1}H NMR spectrum (400 MHz, D_2O) of **P3-Neu5Ac**.

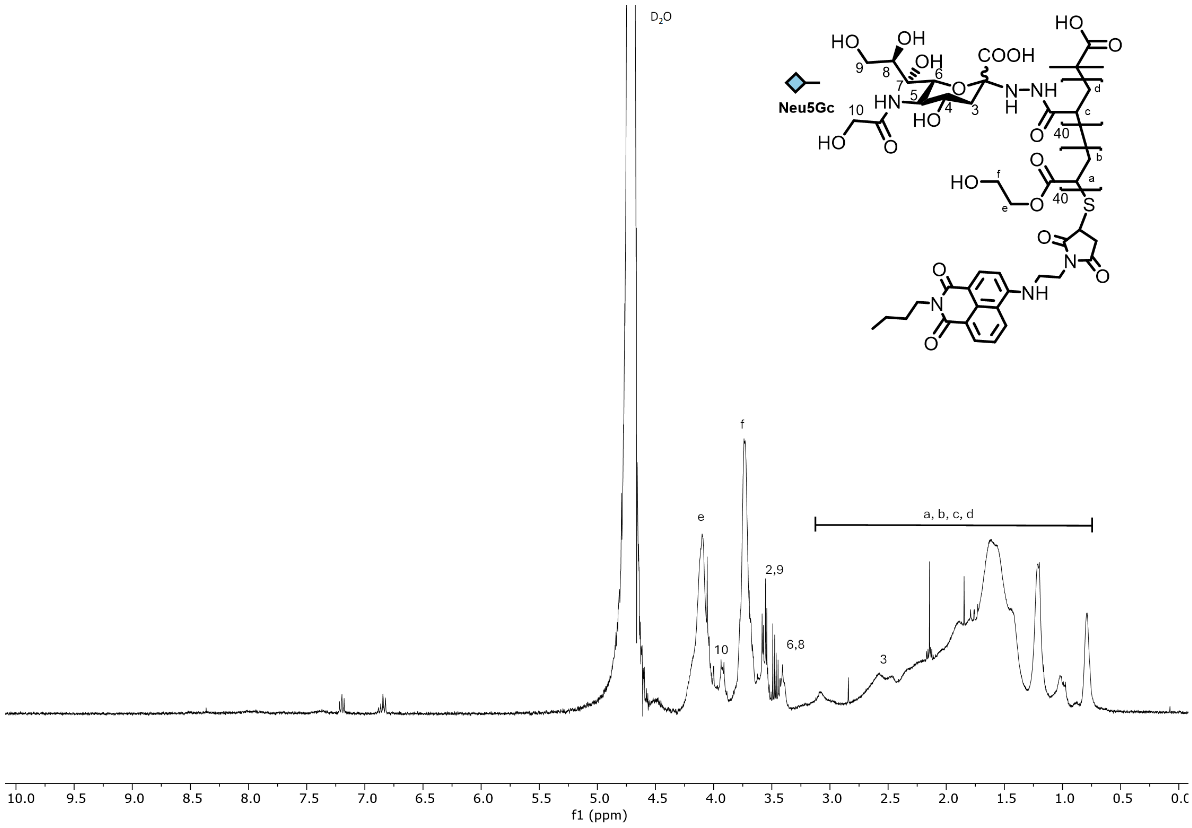

Figure S33 ^{1}H NMR spectrum (400 MHz, D_2O) of **P3-Neu5Gc**.

6. References

1. Lai, J. T.; Filla, D.; Shea, R., Functional Polymers from Novel Carboxyl-Terminated Trithiocarbonates as Highly Efficient RAFT Agents. *Macromolecules,* **2002**, *35* (18), 6754–6756.
2. Metz, N.; Theato, P., Controlled synthesis of poly(acetone oxime acrylate) as a new reactive polymer: Stimuli-responsive reactive copolymers. *Eur. Polym. J. ,* **2007**, *43* (4), 1202–1209.
3. Lim, T.; Ryoo, J. Y.; Jang, M.; Han, M. S., Ligand-free Suzuki–Miyaura cross-coupling with low Pd content: rapid development by a fluorescence-based high-throughput screening method. *Org. Biomol. Chem.,* **2021**, *19* (5), 1009–1016.
4. Peng, H.; Shen, K.; Mao, S.; Shi, X.; Xu, Y.; Aderinto, S. O.; Wu, H., A Highly Selective and Sensitive Fluorescent Turn-on Probe for Al3+ Based on Naphthalimide Schiff Base. *J. Fluorescence,* **2017**, *27* (3), 1191–1200.
5. Mari, C.; Mosberger, S.; Llorente, N.; Spreckelmeyer, S.; Gasser, G., Insertion of organometallic moieties into peptides and peptide nucleic acids using alternative "click" strategies. *Inorg. Chem. Front.,* **2016**, *3* (3), 397–405.
6. Everitt, B. S., *Cluster Analysis*. 5th ed.; Wiley: Chichester, UK, 2011.
7. https://sites.google.com/uw.edu/salipante-lab.
